# Probing Mn Precatalyst Activation through Time-Resolved Spectroscopy: A Quantitative Evaluation of the Effects of CO and PPh_3_ as Coligands on Ultrafast Dynamics and C–C Bond Formation

**DOI:** 10.1021/acs.inorgchem.5c01443

**Published:** 2025-08-12

**Authors:** Benjamin R. O’Donoghue, Stefan Flesch, Eimear Courtney, Shweta Choudhary, Jonathan B. Eastwood, Katrina Mackey, Leticia M. Pardo, Ian P. Clark, Partha Malakar, Gregory M. Greetham, Adrian C. Whitwood, Richard J. Gammons, Gerard P. McGlacken, Ian J. S. Fairlamb, Jason M. Lynam

**Affiliations:** † Department of Chemistry, 8748 University of York, Heslington, York YO10 5DD, U.K.; ‡ School of Chemistry, Analytical & Biological Chemistry Facility, 8795 University College Cork, Cork T12 YN60, Ireland; § Central Laser Facility, STFC Rutherford Appleton Laboratory, Harwell Science and Innovation Campus, Oxfordshire, Didcot OX11 0QX, U.K.

## Abstract

An investigation into the effect of a phosphine coligand on the activation of precatalysts for manganese-catalyzed C–H bond functionalization is reported. Although simple precatalysts [MnBr­(CO)_5_] and [Mn_2_(CO)_10_] are used extensively in these reactions, there is a dearth of alternate precatalyst structures, which has hindered the development of structure–activity relationships. In this work, the effect of substituting a carbonyl ligand for a phosphine ligand is reported. Investigation of the photochemical activation of the precatalyst *fac*-[Mn­(inpy)­(CO)_3_(PPh_3_)] (inpy = cyclometalated 1-(pyridin-2-yl)-1*H*-indole) **3** by time-resolved infrared spectroscopy (TRIR) reveals that light-induced dissociation of a CO ligand occurs preferentially over loss of the phosphine. The ultrafast dynamics of the initially formed solvent complex [Mn­(inpy)­(CO)_2_(toluene)­(PPh_3_)] **9** are described, as is the slower substitution of the coordinated solvent by added pyridine to give [Mn­(inpy)­(CO)_2_(NC_5_H_5_)­(PPh_3_)] **10**. Replacing the pyridine with phenylacetylene again results in the substitution of the metal-bound toluene to give the alkyne complex [Mn­(inpy)­(η^2^-HC_2_Ph)­(CO)_2_(PPh_3_)] **12**. The alkyne undergoes a migratory insertion reaction into the Mn–C bond on a microsecond time scale with a very similar first-order rate constant to [Mn­(inpy)­(CO)_4_], **2**, demonstrating that this key step in Mn-catalyzed reactions is not affected by the presence of the phosphine ligand.

## Graphical abstract

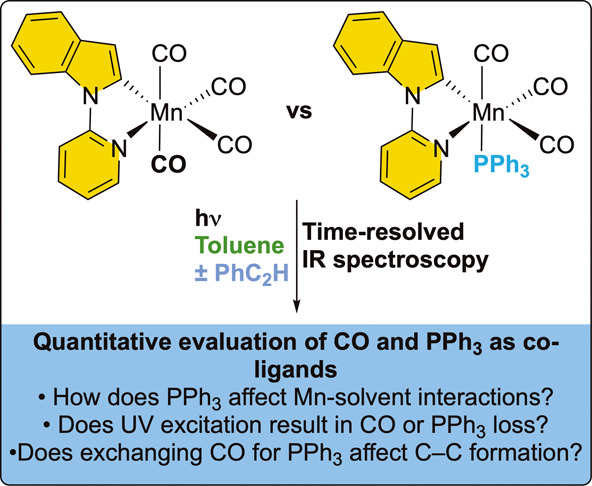

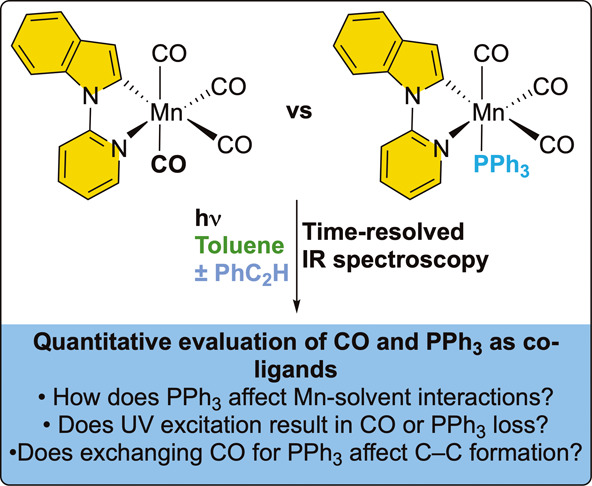

## Introduction

There has been a significant resurgence in the use of metal carbonyl complexes as precatalysts for the modification of complex organic substrates.^1–5^ Manganese carbonyl compounds represent some of the leading examples of this work as simple precatalysts, such as [MnBr­(CO)_5_] and [Mn_2_(CO)_10_], can perform highly selective C–H bond functionalization reactions.^3,6,7^ In general, two reaction outcomes are possible: either the formal insertion of an alkyne (or related electrophile) into a C–H bond or, alternatively, an oxidative coupling can occur to give cyclic products. The precise outcome of these reactions depends on the nature of the two coupling components used; however, a common series of mechanistic steps is thought to occur (Figure 1a). C–H bond activation of the directing group-containing substrate affords manganacycles, which can then undergo a migratory insertion reaction with an unsaturated substrate, such as an alkyne. Protodemetalation leads to the formation of the product arising from formal insertion into the C–H bond, whereas reductive elimination gives the alternative oxidative coupling product.

**Figure 1:**
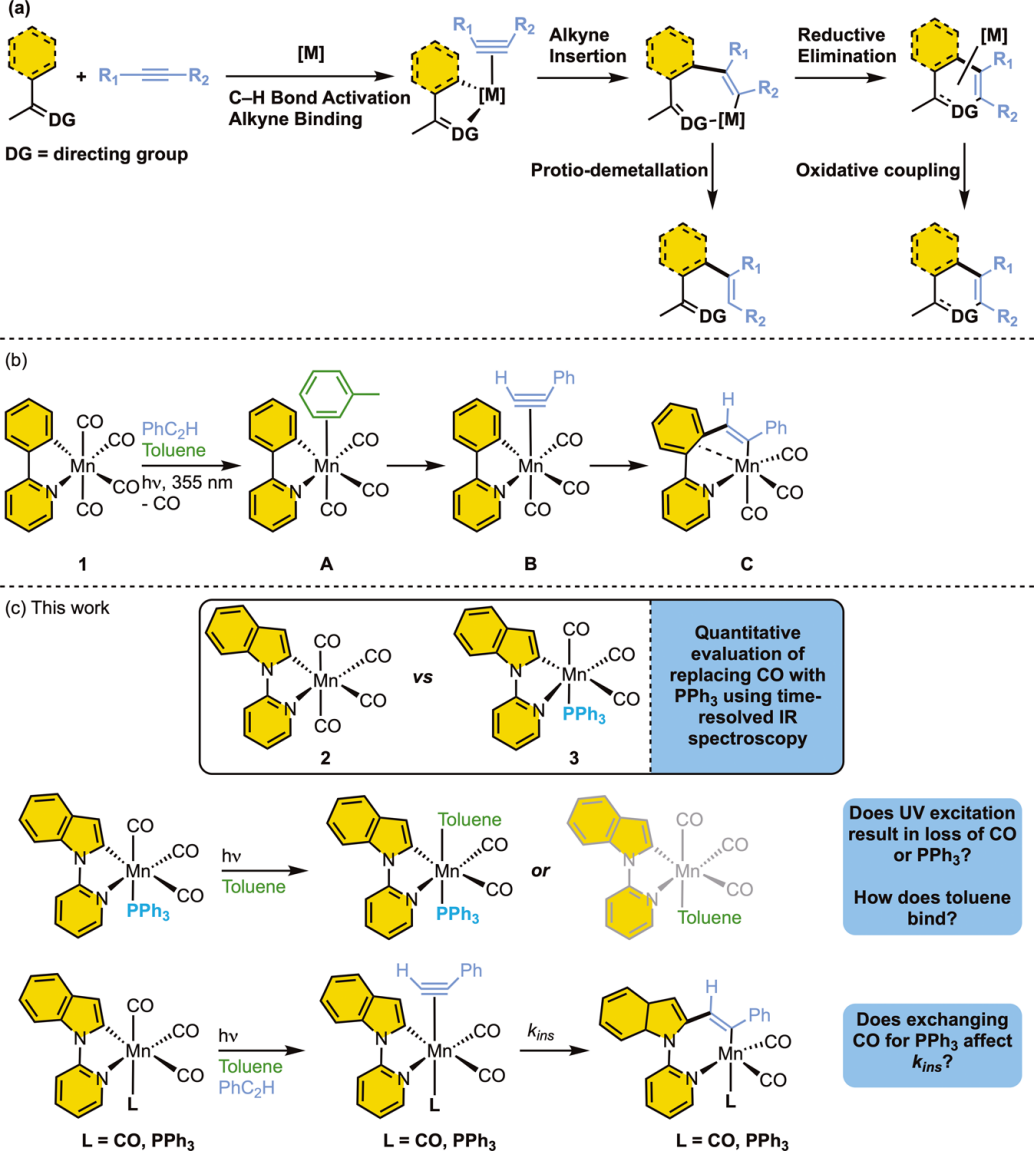
(a) Schematic showing the key steps underpinning Mn-catalyzed C–H bond functionalization reactions. (b) Mechanism derived from previous results obtained by TRIR studies on **1**. (c) Key aims of this work.

Although there have been some developments on the use of different precatalyst structures, e.g. [MnBr­(NCMe)_2_(CO)_3_][Bibr ref8] and [Mn_2_Br_2_(CO)_8_],^9^ both of which appear to deliver the crucial “*fac*-Mn­(CO)_3_” fragment to the reaction mixture, there has been little exploration of the potential role of other coligands to modify and enhance catalytic activity. It is therefore surprising that the effects of introducing phosphorus­(III)-containing ligands into this class of compounds have not been explored, as they represent a diverse and versatile series of ligands in which the steric and electronic properties may be systematically varied.^10,11^ Such an approach could lead to the development of well-defined structure–activity relationships in this class of reactions. With this in mind, it was decided to explore the structural and dynamic effects of incorporating a phosphine ligand into the coordination sphere of an Mn carbonyl compound.

In previous studies, we have shown how insight into the key catalytic processes underpinning Mn-catalyzed C–H bond functionalization reactions may be obtained using time-resolved infrared spectroscopy (TRIR).^12–18^ In these experiments, two laser pulses are used, first a UV or visible photon induces CO-photodissociation from a manganacyclic intermediate such as **1** ^19^ (Figure 1b) to generate an activated complex. The fate of the activated compound is then interrogated using a second infrared laser pulse, which uses the vibrational modes of the remaining carbonyl ligands within the coordination sphere of the metal to report on the resulting speciation and dynamics of the photoproducts. This approach has enabled key mechanistic steps such as solvation (formation of toluene complex **A**), ligand substitution (**A** *→* **B**), and carbon–carbon bond formation (**B** *→* **C**) to be directly observed and quantified on time scales ranging from picoseconds to microseconds. By exploring the effect of a range of different alkynes and manganacycles a comprehensive understanding of the factors affecting this migratory insertion has been obtained.^16^

It was anticipated that a similar approach could be used to investigate the effect of incorporating a phosphine ligand into the coordination sphere of the manganese. Photolysis of a manganacyclic complex [Mn­(C^N)­(CO)_3_(PPh_3_)] (C^N = cyclometalated ligand) could potentially result in either PPh_3_ or CO loss. If the former occurred, then the putative intermediate “[Mn­(C^N)­(CO)_3_]” would be generated, identical to that formed from the photolysis of [Mn­(C^N)­(CO)_4_]. Alternatively, CO loss would give dicarbonyl species “[Mn­(C^N)­(CO)_2_(PPh_3_)]”, and the resulting TRIR spectra would provide information about its interaction with the solvent medium and any reaction substrates. It is also plausible that competing CO and PPh_3_ loss could occur; however, the established photochemistry of [Mn­(C^N)­(CO)_4_] would enable all these possibilities to be readily deconvoluted.

The successful implementation of this strategy is now reported, and a direct comparison of the time-resolved spectroscopic data for the known tetracarbonyl complex [Mn­(inpy)­(CO)_4_], **2**, with the novel phosphine analogue *fac*-[Mn­(inpy)­(CO)_3_(PPh_3_)], **3**, (inpy = cyclometalated 1-(pyridin-2-yl)-1*H*-indole), demonstrates that the selective loss of a CO, rather than a PPh_3_ ligand occurs. The effects of the incorporation of the phosphine ligand on the speciation and dynamics of the resulting light-activated complexes are reported. Notably, the phosphine ligand does not perturb the kinetics of the crucial migratory insertion step between the manganacycle and the alkyne, which underpins the C–C bond formation step in the catalytic cycle.

## Results and Discussion

To investigate the effect of phosphine ligands on the structure and reactivity of manganacycles, the synthesis of triphenylphosphine complex *fac*-[Mn­(inpy)­(CO)_3_(PPh_3_)], **3**, was targeted. Complex **3** was prepared from the reaction of [MnBr­(CO)_4_(PPh_3_)] (**4**) with proligand **5**, 1-(pyridin-2-yl)-1*H*-indole, in the presence of two equivalents of NHCy_2_ in 83% yield (Figure 2a). A single resonance was observed in the ^31^P­{^1^H} NMR spectrum of **3** at δ 53.4, confirming a change in the chemical environment of the coordinated PPh_3_ ligand (compared to δ 41.0 for **4** and δ −5.0 for PPh_3_). Crystals of **3** suitable for study by single-crystal X-ray diffraction were grown by the slow diffusion of hexane into a CH_2_Cl_2_ solution of the complex. The resulting structural determination (Figure 2c) confirmed the presence of the cyclometalated inpy ligand and a *fac*-geometry of carbonyl ligands around the Mn atom.

**Figure 2:**
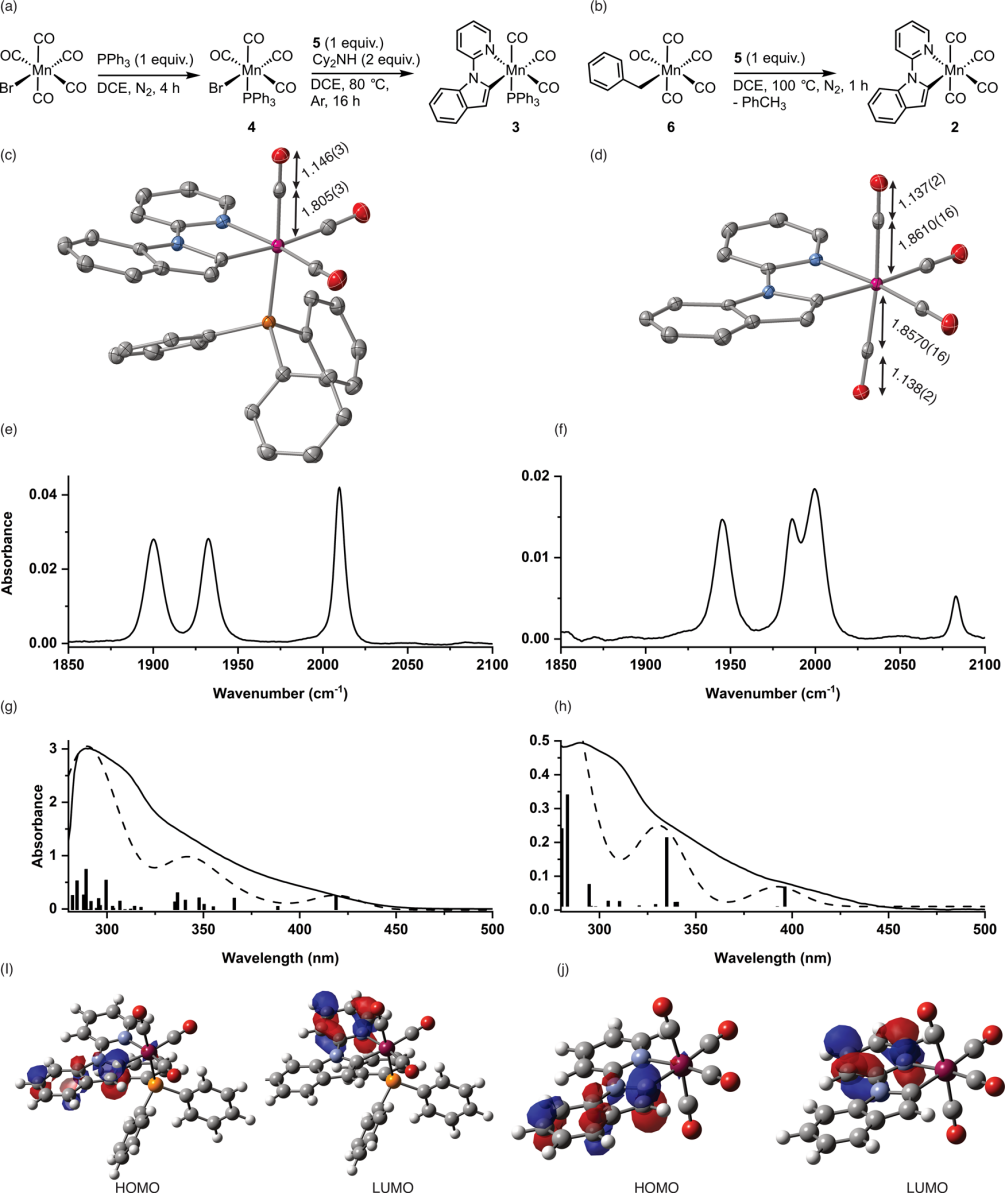
(a) Preparation of **3** from [MnBr­(CO)_5_], (b) preparation of **2** from [MnBn­(CO)_5_], (c) structure of **3** determined by single-crystal X-ray diffraction, and (d) structure of **2** determined by single-crystal X-ray diffraction. In both cases, the thermal ellipsoids are shown at the 50% probability level, and hydrogen atoms have been omitted for clarity. Carbon atoms are shown in gray, oxygen in red, nitrogen in blue, phosphorus in orange, and manganese in purple. Selected bond lengths are shown in Å. (e) Infrared spectrum of **3** in the metal carbonyl region recorded in toluene solution (0.502 mM, path length 200 μm). (f) Infrared spectrum of **2** in the metal carbonyl region recorded in toluene solution (0.502 mM, path length 200 μm). (g) Electronic spectrum of **3** recorded in toluene solution (0.165 mM, path length 1 cm). (h) Electronic spectrum of **2** recorded in toluene solution (0.175 mM, path length 2 mm). For (f) and (g), the bars are the DFT-predicted electronic transitions at the b3lyp/def2-TZVPP//bp86/sv­(p) level with COSMO solvent correction in toluene, scaled by relative oscillator strength, and the dashed line is a spectrum generated from these calculated transitions by fitting to Gaussian functions with a line width of 15 nm. (i) HOMO and LUMO of **3.** (j) HOMO and LUMO of **2**.

To investigate the effect of the PPh_3_ ligand on the structure, the known complex, *fac*-[Mn­(inpy)­(CO)_4_], **2**, was prepared using an alternative route from the reaction of **5** with [Mn­(CH_2_Ph)­(CO)_5_], giving **2** in 90% yield (Figure 2b). Crystals of **2** ^20^ were grown identically to **3**, and results from the structure determination are shown in Figure 2d. Comparison of the structures of **2** and **3** demonstrated that the replacement of a phosphine ligand for a carbonyl group had a statistically negligible effect on the Mn-inpy bond metrics as well as for the two CO groups in the same plane, as this is a cyclometalated ligand. In contrast, the Mn–C bond for the carbonyl ligand *trans* to PPh_3_ in **3** was notably shortened (1.805(3) Å) when compared to that of the two mutually *trans* carbonyl ligands (1.8570(16) and 1.8610(16) Å). This is consistent with the phosphine ligand being a poorer π-acceptor than the CO.

To aid with an evaluation of the photoproducts formed from irradiation of complexes **2** and **3,** a discussion of their ground state infrared spectra (Figure 2e,f, respectively) is pertinent. In the metal–carbonyl stretching region, **2** exhibits four bands at 1945, 1986, 1999, and 2083 cm^–1^, which may be assigned to *B*_2_, *A*_1_, B_1_, and *A*_1_ modes, respectively, in a pseudo-*C*_*2v*_ symmetric complex of the general type [Mn­(C^N)­(CO)_4_].^21,22^ In contrast, complex **3** exhibits three bands at 1900, 1932, and 2010 cm^–1^, which are assigned to two asymmetric (1900 and 1932 cm^–1^) and one symmetric (2010 cm^–1^) MC≡O stretching modes. The fact that all three bands have approximately the same intensity is consistent with a *facial* arrangement of carbonyl ligands at Mn as in the *meridional* case one band would be considerably weaker.^21,23^

The photochemical activation of **2** and **3** was then explored using time-resolved infrared spectroscopy. In this experiment, a UV/vis laser pulse is used to activate the substrate, and the structural and dynamic changes are interrogated with a subsequent probe in the IR. The nature of the resulting photoproducts was investigated through changes to the position and intensity of the bands due to the vibration of the MC≡O groups. The data are displayed as difference spectra with negative peaks due to species consumed on photolysis (in this work corresponding to the ground state IR spectrum of **2** or **3**), whereas the positive features are the products formed following the absorption of the probe light. The experiments were performed on the LIFEtime spectrometer in the ULTRA facility (Rutherford Appleton Laboratory, UK) using the time-resolved multiple probe (TR^M^PS) method, which, by synchronization of the pump (1 kHz) and probe (100 kHz) lasers, enables data to be collected with pump–probe delays between 1 ps and 1 ms.

The electronic spectra of complexes **2** and **3** were acquired to select an appropriate excitation wavelength for the pump pulse in the TRIR experiment. As shown in Figure 2g (**3**) and Figure 2h (**2**), the spectra recorded in toluene solution show broad absorption bands at λ < 500 nm, with **3** showing a lower energy absorption tail than **2** at 450 nm. The spectra were simulated using time-dependent density functional theory (TD-DFT), which reproduced the key features of the spectra, including the fact that the lowest energy transition of **3** was red-shifted when compared to that of **2**. The TD-DFT calculations indicated that in both cases, these lowest energy transitions had greater than 95% HOMO → LUMO character. In both cases, the HOMO was based on the indole moiety of the inpy ligand with the LUMO on the pyridine (Figure 2i,j). Therefore, these transitions are best viewed as having intraligand charge transfer character. Based on these data, an excitation wavelength of 400 nm was selected for the TRIR experiments.

To provide reference data for the modifications induced on incorporation of the PPh_3_ ligand into the coordination sphere of the metal, the behavior of the known tetracarbonyl complex, [Mn­(inpy)­(CO)_4_], **2**, was explored using TRIR. By analogy with previous work on [Mn­(ppy)­(CO)_4_], **1**, (ppy = cyclometalated 2-phenylpyridine), it was anticipated that CO-dissociation from **2** in toluene solution would produce a labile solvent complex [Mn­(inpy)­(CO)_3_(toluene)], **7**, which would undergo reaction with dinitrogen or trace amounts of water.^21,24^ Therefore, an excess of pyridine was added to the toluene solution to ensure that the resulting complexes would be stable, enabling their characterization. The spectra resulting from these experiments are shown in Figure 3.

**Figure 3:**
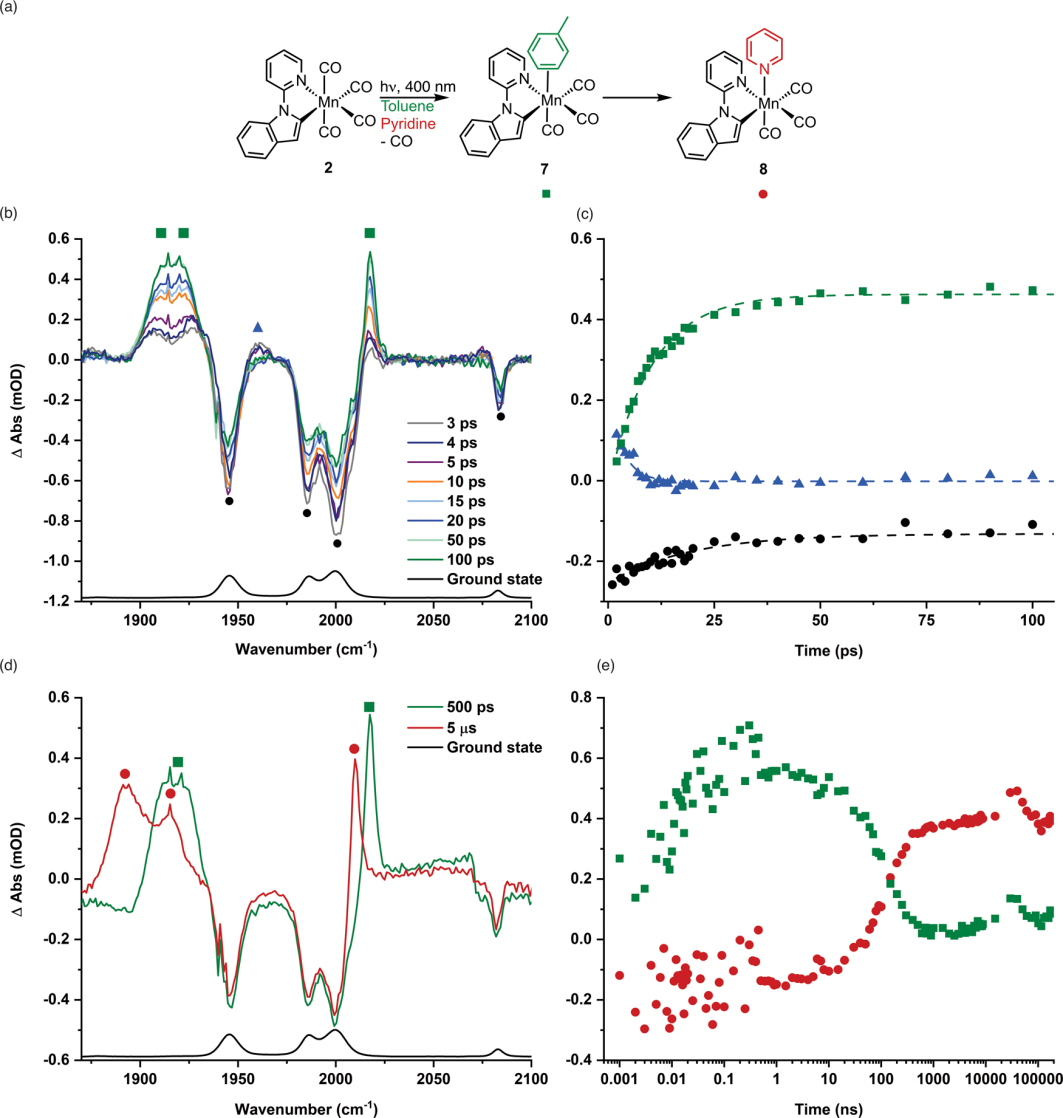
(a) Reaction scheme showing the formation of the products from photolysis of **2** in a toluene solution containing an excess of pyridine. (b) TRIR spectra of [Mn­(inpy)­(CO)_4_] in toluene solution containing pyridine recorded with selected pump–probe delays up to 100 ps. (c) Kinetic trace showing the time course for the formation of **7** using the change in intensity of the peak at 2017 cm^–1^ (green squares, the dashed line is a fit with τ = (10.3 ± 0.5) ps and *R* ^2^ = 0.984), loss of 2* using the change in the intensity of the peak at 1961 cm^–1^ (blue triangles, the dashed line is a fit with τ = (3.2 ± 0.6) ps and *R* ^2^ = 0.801), and bleach recovery for **2** using the change in the intensity of the peak at 1986 cm^–1^ (black circles, the dashed line is a fit with τ = (19.2 ± 3.2) ps, *R* ^2^ = 0.873). (d) TRIR spectra of [Mn­(inpy)­(CO)_4_] in toluene solution containing pyridine recorded with pump–probe delays of 500 ps and 5 μs. (e) Kinetic trace with a logarithmic time axis showing the time course for the conversion of **2** to **7** and **8** using the change in the intensity of the peak at 2017 cm^–1^ (green squares) and 2010 cm^–1^ (red circles).

The spectra all exhibited four negative peaks corresponding to the ground state spectrum of **2**, confirming that the complex was consumed on photolysis. At early times (<20 ps), a number of positive features were observed, including a peak at 1961 cm^–1^ (purple triangle, Figure 3b). The species responsible for this peak had a short lifetime (ca. 3 ps,^25^ Figure 3c^26^). Again, by analogy to the related feature observed in the spectra of **1**, the species responsible for these features was assigned to an electronically excited state of **2**, 2*. Several other positive features were also observed. At early times, two bands were observed at 1909 and 1924 cm^–1^, which over the course of ca. 10 ps sharpened and became a single broad feature centered at 1915 cm^–1^. A related peak at 2018 cm^–1^ was also observed (green squares, Figure 3b). These bands were assigned to the toluene complex [Mn­(inpy)­(CO)_3_(toluene)], **7**. This complex is formed by the light-induced loss of CO, and the resulting vacant coordination site is then occupied by the solvent. The observed change in the line shape may be due to two factors. As the energy of the excitation photon is greater than the Mn–CO bond dissociation energy, the primary photoproducts are formed in an excited vibrational energy level. The subsequent cooling to the ν = 0 energy level results in a sharpening of the observed bands and, due to the anharmonic nature of the vibrational energy well, a shift to higher frequency.^27,28^ Although the bands for **7** do sharpen, the band at 1924 cm^–1^ shifts to a lower frequency. Therefore, a second possible explanation for this behavior is that the initial binding of the toluene ligand to the metal occurs in a kinetically controlled manner, which is then followed by a rearrangement to the more thermodynamically preferred form. Indeed, in the case of [Mn­(ppy)­(CO)_4_] in THF solution, the solvent initially binds in a C–H (σ) fashion before rearranging over the course of ca. 20 ps to the more thermodynamically favorable *O*-bound form.^21^ It should also be noted that the four bleach bands for **2** also show some recovery on a picosecond time scale—this may be due to a combination of relaxation from 2* to **2** and geminate recombination of CO to give **2**, again in a higher vibrational energy level. The observed decrease in the intensity of the negative bands is associated with the vibrational cooling from these excited vibrational states.^27^

Over the course of ca. 100 ns, the bands for **7** decreased in intensity to be replaced by peaks at 1893, 1916, and 2010 cm^–1^. The large shift in the MC≡O vibrational frequencies to lower energy is consistent with the displacement of the toluene ligand and formation of [Mn­(inpy)­(CO)_3_(NC_5_H_5_)], **8** (red circles, Figure 3d) by the pyridine, a better donor ligand. The loss of the peaks for **7** and growth of the peaks for **8** (Figure 3e) were successfully fitted to single exponential functions with rate constants of (7.74 ± 0.55) × 10^6^ s^–1^ and (7.04 ± 1.92) × 10^6^ s^–1^, respectively. The successful fit is consistent with the reaction being governed by pseudo first-order kinetics as the pyridine is in large excess.

These data therefore present a picture in which ultrafast light-induced loss of a CO ligand from **2** occurs. The resulting putative unsaturated complex “[Mn­(inpy)­(CO)_3_]” then undergoes statistically controlled coordination to the reaction solvent, toluene. As the solvent is the most abundant component of the reaction, there is a kinetic preference for manganese to initially bind to the toluene rather than the more dilute pyridine, and therefore, **7** is formed. Toluene is, however, a poor or “token” ligand^29^ for the metal and undergoes rapid substitution by the added pyridine to give **8**.

The TRIR experiment was then repeated in an identical fashion, but **2** was substituted by **3**. At all positive pump–probe delays, three strong negative bands were observed, corresponding to the ground state spectrum of **3**, confirming that the complex had been consumed on photolysis. At early times (<10 ps), a strong peak was observed at 1946 cm^–1^ (purple triangle, Figure 4b), which decayed with a lifetime of 11 ps. Two other peaks were observed to grow in intensity at 1850 and 1922 cm^–1^ with lifetimes of 12 and 10 ps, respectively. The observation of two bands of equal intensity confirmed that CO loss, rather than PPh_3_ loss, had occurred, and a complex with two mutually *cis* carbonyl ligands had been formed. This is consistent with the loss of a carbonyl ligand from **3** and the formation of *fac*-[Mn­(inpy)­(CO)_2_(PPh_3_)­(toluene)], **9** (Figure 4a). If phosphine loss, rather than CO loss, was occurring, then *fac*-[Mn­(inpy)­(CO)_3_(toluene)], **7**, would be obtained, which is the same species identified on photolysis of **2** (Figure 3b). As no peaks attributable to **7** were observed, CO loss is the dominant photochemical process. Over the course of ca. 10 ps, the two peaks for **9** increased in intensity and showed small shifts in band positions which, by analogy to the behavior of [Mn­(inpy)­(CO)_3_(toluene)] on a similar time scale, was assigned to an initial unselective toluene-binding event, followed by a reorientation to the most thermodynamically preferred binding mode.

**Figure 4:**
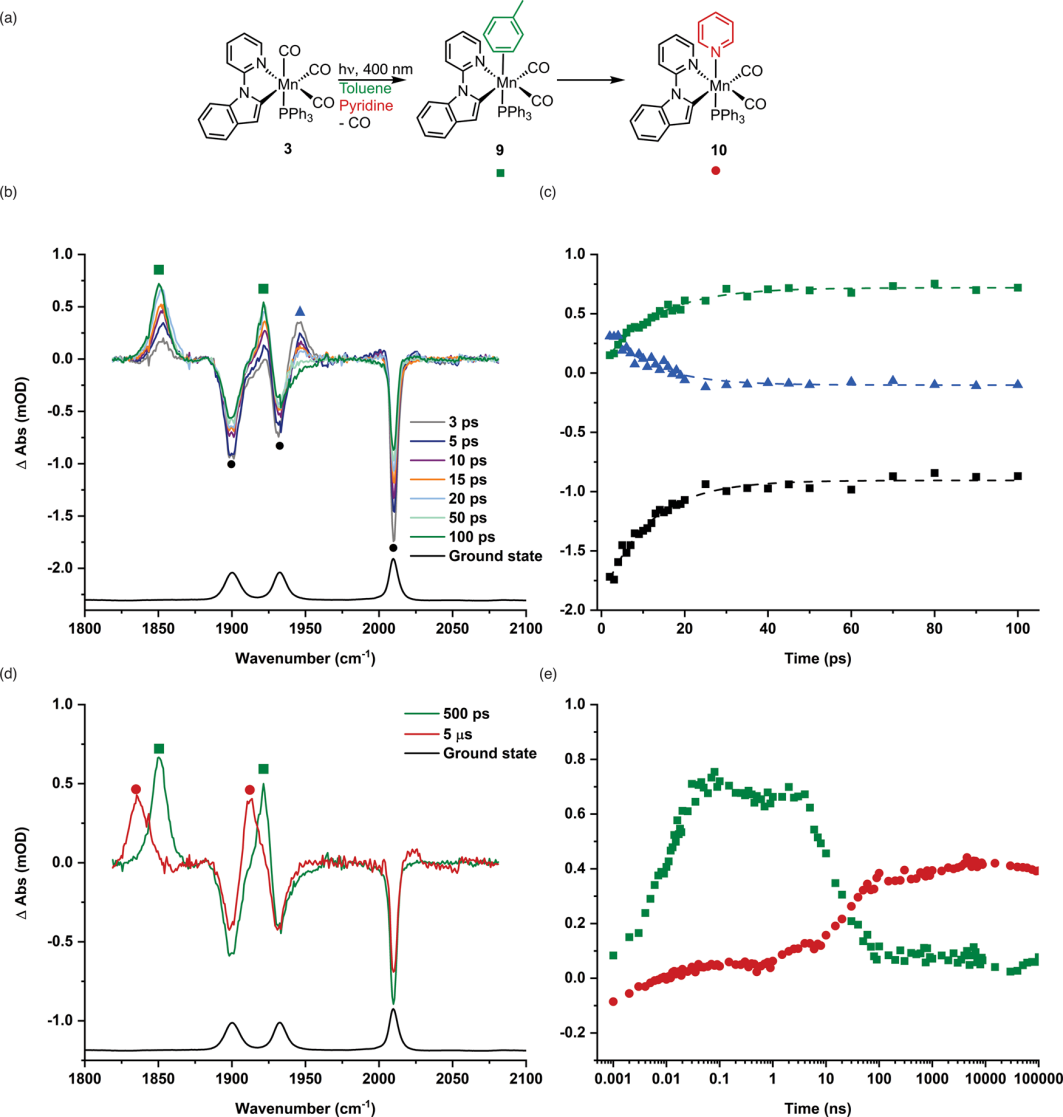
(a) Reaction scheme showing the formation of the products from the photolysis of **3** in toluene solution. (b) TRIR spectra of [Mn­(inpy)­(CO)_3_(PPh_3_)] **3** in toluene solution recorded with selected pump–probe delays up to 100 ps. (c) Kinetic trace showing the time course for the formation of **9** using the change in the intensity of the peak at 1850 cm^–1^ (green squares, the dashed line is a fit with τ = (12.4 ± 0.2) ps and *R* ^2^ = 0.978), loss of 3* using the change in intensity of the peak at 1946 cm^–1^ (blue triangles, the dashed line is a fit with τ = (10.9 ± 0.3) ps and *R* ^2^ = 0.932) and bleach recovery for **3** using the change in intensity of the peak at 2011 cm^–1^ (black squares, the dashed line is a fit with τ = (11.1 ± 0.2) ps and *R* ^2^ = 0.974). (d) TRIR spectra of [Mn­(inpy)­(CO)_4_(PPh_3_)] in toluene solution in the presence of pyridine recorded with pump–probe delays of 500 ps and 5 μs. (e) Kinetic trace with a logarithmic time axis showing the time course for the conversion of **3** to **9** and **10** using the change in intensity of the peak at 1850 cm^–1^ (green squares) and 1835 cm^–1^ (red circles).

The nature of the species responsible for the peak at 1946 cm^–1^ requires further comment. By analogy to the previously reported photochemistry of **1** and [Cr­(bpy)­(CO)_4_],^21,23^ this species could be assigned to the electronic excited state of **3**, 3*, and its decay corresponds to relaxation to the electronic ground state of **3**. Consistent with this explanation, the three bleach bands for **3** all recovered with similar time constants (1900 cm^–1^, τ = 10 ps; 1933 cm^–1^ τ = 9 ps, 2011 cm^–1^ τ = 11 ps). However, the fact that **9** appears to also grow with the same time constant as the loss of 3* cannot be ignored. This may, of course, be coincidental, as the growth of the peaks for **7** had essentially the same time constant, but state 2* is much shorter-lived. Although photochemical CO dissociation typically occurs on a femtosecond time scale,^30^ we cannot exclude the possibility that 3* may undergo relaxation to the electronic ground state to reform **3** with a competing delayed CO release.^31^

Over the course of ca. 100 ns, the intensity of the peaks due to **9** decreased to be replaced by new peaks at 1835 and 1912 cm^–1^. In a similar vein to the observation when using complex **2**, the shift in the band position to lower energy is consistent with the added pyridine, substituting the coordinated toluene solvent, giving *fac*-[Mn­(inpy)­(CO)_2_(PPh_3_)­(NC_5_H_5_)], **10**. Complex **10** grew with a pseudo-first-order rate constant of (4.60 ± 0.75) × 10^7^ s^–1^, commensurating with the corresponding rate constant for the loss of **9**, (4.80 ± 0.67) × 10^7^ s^–1^.

A series of calculations using density functional theory (DFT) was performed to provide further insights into the observed behavior of the complexes. In order to evaluate the Mn-toluene interactions, a series of geometry optimizations was undertaken on different metal–solvent distances in complexes **7** and **9**. In both cases, it was found that the most stable form had the toluene binding in a π-fashion to the Mn, **7**(π) and **9**(π) (Figure 5a) and that a C–H­(σ) type interaction between the metal and the methyl group of the toluene, **7**(CH_3_-σ) and **9**(CH_3_-σ), was at higher energy. The same observation was obtained with the toluene complex [Mn­(ppy)­(CO)_3_(toluene)], **11**, obtained from [Mn­(ppy)­(CO)_4_], **1**.^21^ However, in the phosphine-containing case, the difference in free energy between the two potential binding modes (+7 kJ mol) was slightly lower compared to **7** (+16 kJ mol^–1^). Moreover, examination of the calculated geometries for the toluene complexes **7**, **9**, and **11** demonstrated that, regardless of binding mode, Mn–solvent interactions increased in length with **11** < **7** < **9** (Figure 5c,d). The difference between **7** and **9** may be interpreted as a decrease in the Lewis acidity of the metal when changing from a CO to PPh_3_ ligand, where comparing **9** to **11** may reflect a similar change but caused by changing the inpy ligand to ppy. In the case of the phosphine-containing complexes, an additional binding mode **9**(CH-σ) was located as a minimum, although the corresponding structural optimization for **7** resulted in **7**(π).

**Figure 5:**
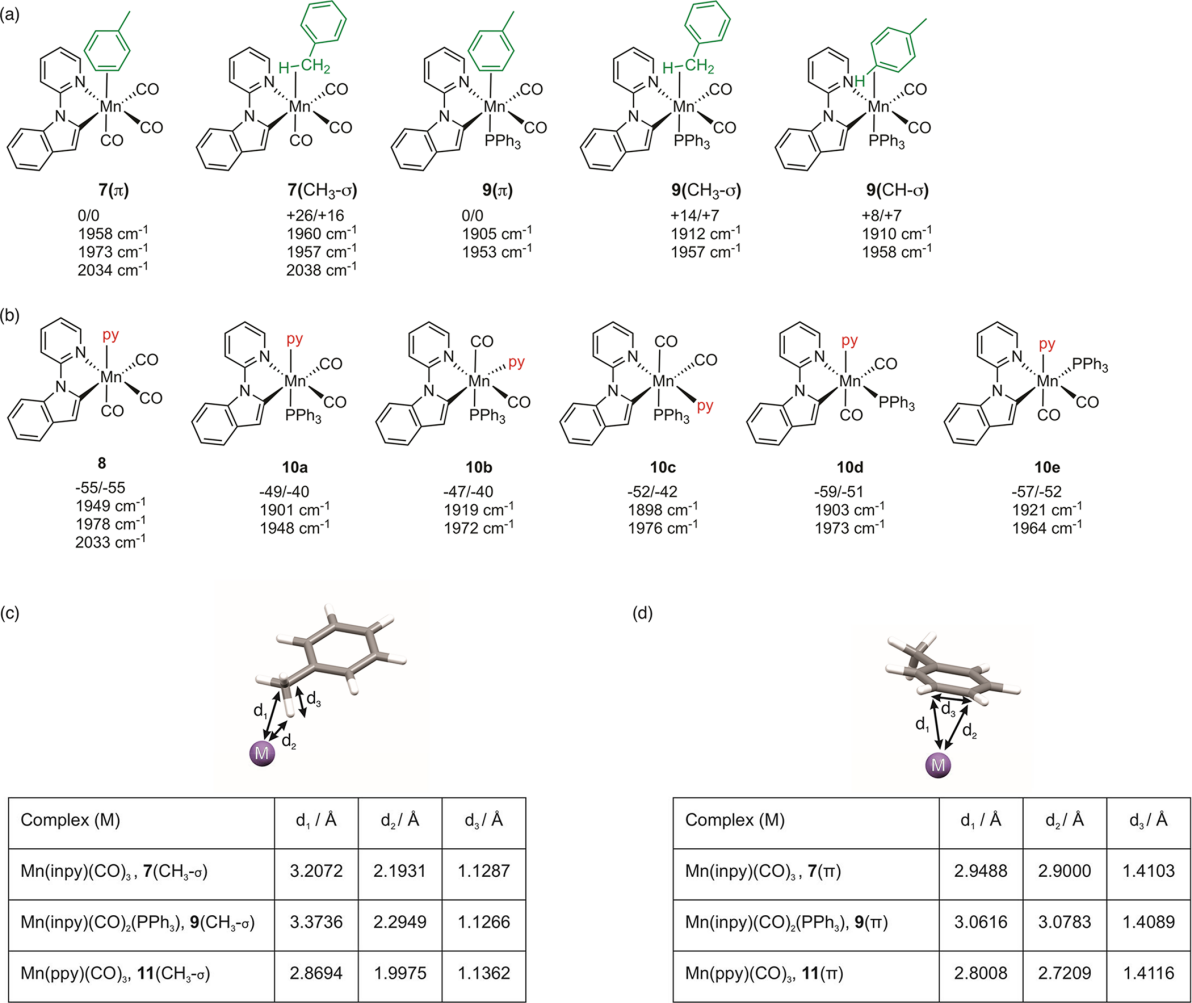
(a) DFT-calculated relative energies and uncorrected harmonic vibrational frequency for ν­(CO) bands in toluene complexes **7** and **9**. (b) DFT-calculated relative energies and uncorrected harmonic vibrational frequency for ν­(CO) bands for pyridine solvent complexes **8** and **10**. (c) Tabulated bond metrics for CH_3_-σ-bound toluene complexes, with the manganese atom shown as a purple sphere. The remainder of the ligands has been omitted for clarity. (d) Tabulated bond metrics for π-bound toluene complexes, with the manganese atom shown as a purple sphere. The remainder of the ligands have been omitted for clarity. Energies are zero-point energy-corrected electronic energies (first entry) and Gibbs energies at 298 K in kJ mol^–1^ (second entry) at the D3­(BJ)-pbe0/def2-TZVPP//bp86/def2-sv­(p) level of theory with COSMO solvent correction in toluene.

In contrast to the data obtained for **2**, which unambiguously demonstrated the formation of a *fac*-Mn­(CO)_3_ unit in **7** and **8**, the spectroscopic data for **9** and **10** only demonstrated that a complex with two mutually *cis* carbonyl ligands is formed. Five possible isomers of *cis*-[Mn­(inpy)­(CO)_2_(PPh_3_)­(NC_5_H_5_)], **10a–e,** are possible (Figure 5b). In the case of **10a**, the two CO ligands are in the same plane as the inpy ligand, and the calculations predict that the two vibrational modes for the complex should be at lower energy than those for all possible forms of the toluene complex (Figure 5a). However, in the other isomers, one CO ligand will be *trans* to either a phosphine (**10b**/**10c**) or a pyridine (**10d**/**10e**) ligand. The calculations predict in all cases that the symmetric stretching mode will be at an energy *higher* than the corresponding toluene complex, which is inconsistent with the experimental observations. Therefore, complex **10** is assigned structure **10a** and supports a picture in which the CO ligand that is *trans* to PPh_3_ is lost on photolysis. This must be a kinetic effect, as the calculations indicate that isomers **10d** and **10e** are at a lower energy than **10a**. Attempts to perform a similar analysis on the different geometric isomers of toluene complex **9** showed a significant method dependence. In the absence of a dispersion correction, optimization led to toluene dissociation with concomitant formation of C–H agostic complexes, whereas when this correction was employed, π-bound toluene complexes could be obtained (see ESI for further discussion).

To investigate the effect of the phosphine ligand on the key C–C bond formation step that underpins Mn-catalyzed C–H bond functionalization reactions, TRIR spectra of a toluene solution of [Mn­(inpy)­(CO)_3_(PPh_3_)] **3** with added PhC_2_H were acquired. At early times (<1 ns), the spectra appeared identical to those recorded in toluene alone, which is consistent with the initial binding event to the Mn following CO loss being kinetically controlled: there is more toluene in the sample than PhC_2_H. Over the course of ca. 10 ns, the two bands for the toluene complex **9** decreased in intensity to be replaced by two new features at 1868 and 1945 cm^–1^. By analogy to the data from related studies,^12,15–17^ this species was assigned to [Mn­(inpy)­(η^2^-HC_2_Ph)­(CO)_2_(PPh_3_)] **12**—this is supported by the fact that the two bands at 1868 and 1945 cm^–1^ were of similar intensity, consistent with the presence of two mutually *cis* carbonyl ligands and notably at a higher frequency than the corresponding pyridine complex. This may be rationalized on the basis of the alkyne ligand being a good π-acceptor. It is instructive to compare these peaks to those obtained previously for [Mn­(inpy)­(η^2^-HC_2_Ph)­(CO)_3_] **13**.^12^ The band positions are notably different than those in the case of the PPh_3_-containing complexes, again confirming that photochemical CO loss predominates over phosphine loss. In addition, the peaks for [Mn­(inpy)­(η^2^-HC_2_Ph)­(CO)_2_(PPh_3_)], **12**, were ca. 50 cm^–1^ lower in energy, reflecting the fact that PPh_3_ is a poorer π-acceptor than CO.

Over the course of a further 50 μs, the bands due to [Mn­(inpy)­(η^2^-HC_2_Ph)­(CO)_2_(PPh_3_)] **12** decreased in intensity to be replaced by features at 1852 and ca. 1930 cm^–1^, the latter overlapping with the bleach band for **3**. Based on the data from related systems,^12,16^ this transformation was assigned to the migratory insertion of the alkyne into the Mn–C bond to give manganacycle **14**. The loss of the peak at 1866 cm^–1^, corresponding to **12**, was fitted to a first-order kinetic model with *k* = (1.37 ± 0.16) *×* 10^4^ s^–1^, whereas the formation of **14** (from the growth of the peak at 1854 cm^–1^) had a similar rate constant of (1.99 ± 0.44) *×* 10^4^ s^–1^. This is essentially identical to that reported rate constant for the migratory insertion of phenylacetylene in the analogous compound [Mn­(inpy)­(η^2^-HC_2_Ph)­(CO)_3_] **13**, (2.00 ± 0.08) *×* 10^4^ s^–1^.

The structures of three different potential geometric isomers of complex **12**, based on a *fac* Mn-(CO)_2_(PPh_3_) framework, and their subsequent migratory insertion reaction through **TS**_**12–14**_ to form complex **14** were calculated by DFT (Figure 6c). These calculations demonstrate that the pathway based on complex **12a** (in which the phosphine ligand was *trans* to the alkyne) to give **14a** was the preferred pathway both kinetically and thermodynamically. Notably, complex **12a** was the lowest energy isomer of the three alkyne complexes evaluated and had the same arrangement of carbonyl and phosphine ligand with respect to the metalacyclic ligand as that predicted for pyridine complex **10**. In addition, the free energy of activation of the alkyne insertion in this pathway through **TS**_**12a**–**14a**_ was found to be 44 kJ mol^–1^, whereas for [Mn­(inpy)­(η^2^-HC_2_Ph)­(CO)_3_] **13,** it was 48 kJ mol^–1^. This is consistent with the experimental rate constants for the migratory insertion of PhC_2_H in the two complexes being statistically identical. Interestingly, however, the driving force for the reaction was greater (103 kJ mol^–1^) for the formation of **14a** when compared to the tricarbonyl analogue (86 kJ mol^–1^).

**Figure 6:**
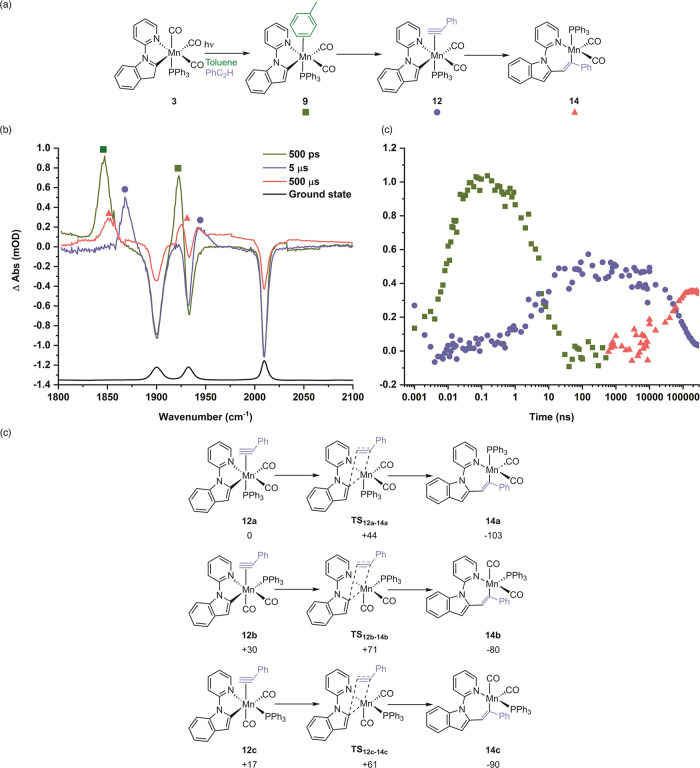
(a) Reaction scheme showing the formation of the products from the photolysis of **3** in toluene solution in the presence of PhC_2_H. (b) TRIR spectra of [Mn­(inpy)­(CO)_3_(PPh_3_)] **3** in toluene solution in the presence of PhC_2_H recorded with pump–probe delays of 500 ps, 5 and 500 μs. (c) Kinetic trace with a logarithmic time axis showing the time course for the conversion of **3** to **9**, **12**, and **14** using the changes in intensity at 1848 cm^–1^ (green squares), 1867 cm^–1^ (blue circles), and 1848 cm^–1^ (orange triangles), respectively. (c) DFT pathways for the migratory insertion reaction of three different isomers of complex **12.** Relative energies (Gibbs energies in kJ mol^–1^ at 298 K) are at the D3­(BJ)-pbe0/def2-TZVPP//bp86/def2-sv­(p) level of theory with COSMO solvent correction in toluene relative to **12a**.

Overall, these data demonstrate that the rate of the migratory insertion reaction is broadly unaffected by the change in the coligand from CO to PPh_3_ and may reflect our previous observation that it is the nature of the metallacycle (inpy in this case) and alkyne (PhC_2_H) that dictates the rate of the migratory insertion reaction.

## Conclusions

The key findings from this study are shown in Figure 7 and highlight that photolysis of [Mn­(inpy)­(CO)_3_(PPh_3_)] at 400 nm in toluene solution results in CO, rather than PPh_3_ photodissociation. The products obtained are consistent with the CO that was *trans* to the phosphine ligand being lost. The data also demonstrate that the ultrafast dynamics of these complexes are complex, with evidence for electronic excited states 2* and 3* obtained, while the presence of the phosphine ligand appears to prolong the lifetime of 3* (11 ps) when compared to 2* (3.2 ps). In both cases, the bleach bands arising from consumption of **2** and **3** show some recovery on time scales <20 ps. This may be due to two mechanisms: first, the relaxation of the electronically excited states would regenerate the ground state; alternatively, this bleach recovery may be due to vibrational relaxation. Here, primary geminate recombination of the photodissociated CO with the Mn prior to it leaving the solvent cage yields the complexes in vibrationally excited states with *v* > 0, and the bleach recovery corresponds to relaxation to the vibrational ground state.^27,30^ In the case of **3**, it appears that the former process dominates as the intense band for 3* decays with the same time constant as the bleach recovery and reformation of **3** (11 ps). In the case of **2**, the extent of the bleach recovery is much less than in **3**; it has a greater time constant (19 ps), and the proportion of 2* formed is proportionally less. Therefore, for **2,** the dominant process responsible for the recovery of the ground state is proposed to be the primary geminate recombination of CO. Secondary geminate recombination (i.e., addition of a CO that has escaped the solvent cage and is undergoing diffusion-controlled processes) is not expected to occur on these time scales, as it will depend on the product of the concentrations of the Mn-containing photoproduct and CO, both of which are expected to be low.

**Figure 7:**
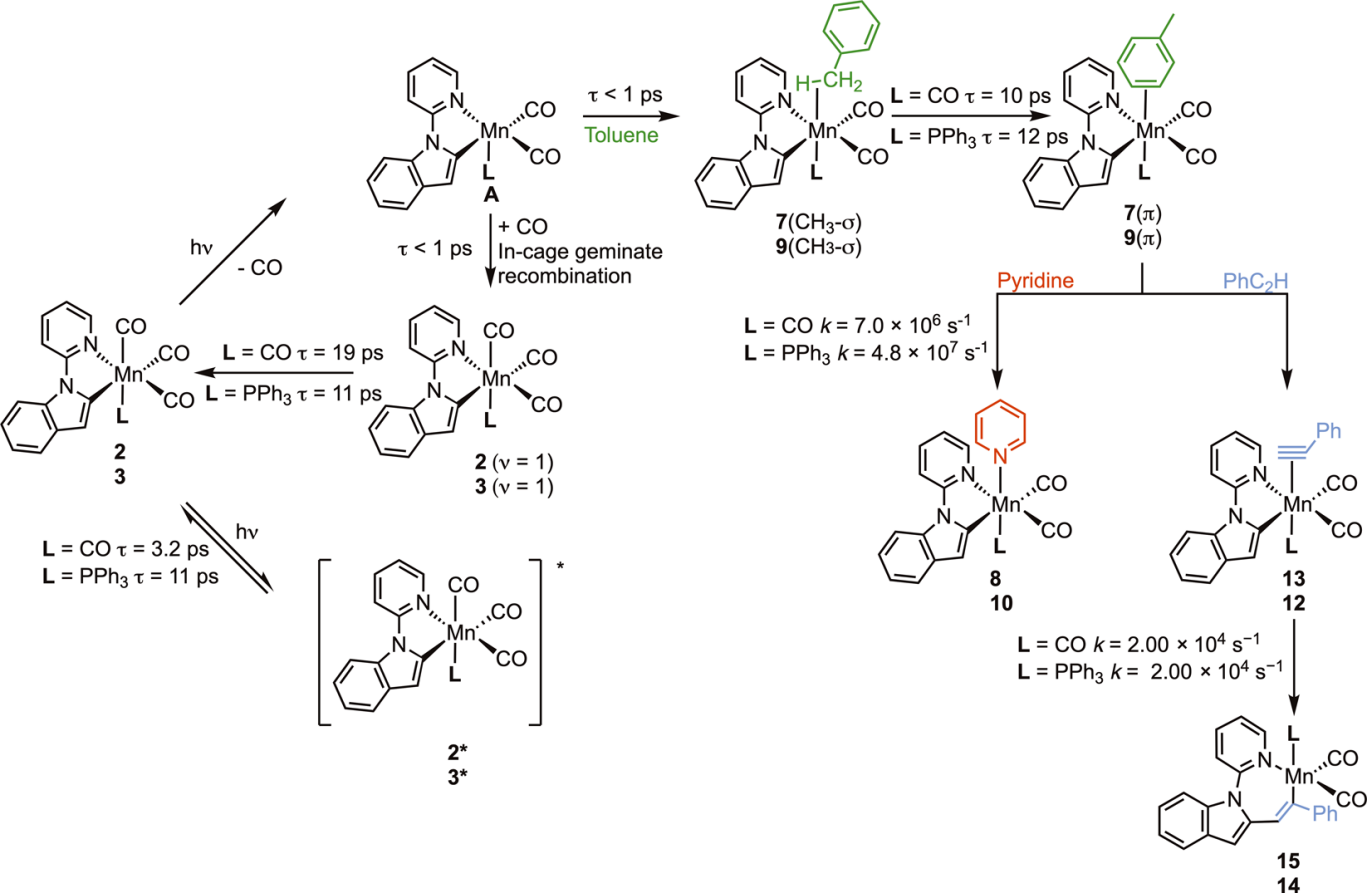
Summary of the key findings from this work. Numerical labels are given for each complex with **L** = CO (top) and **L** = PPh_3_ (bottom). Time constants and rate constants have been quoted to two significant figures: data with appropriate confidence limits are given in the manuscript.

Additional early time dynamics were observed for both **2** and **3** that are assigned to the kinetically controlled binding of the formally unsaturated complex **A** (Figure 7) with the toluene solvent medium and the formation of **7**(CH_3_-σ) and **9**(CH_3_-σ), respectively. A rearrangement to the more thermodynamically stable complexes **7**(π) and **9**(π) occurs with time constants of 10 and 12 ps, respectively. Such dynamic metal–solvent interactions have previously been observed in ether, DMSO, and alcohol solvents.^21,32^ An alternative explanation for these observations is that the spectroscopic changes are due to the initial solvation event of unsaturated complex **A**. Although our calculations cannot discern the difference between these two possibilities, the predicted vibrational frequencies of **A** and **9**(CH_3_-σ) are very similar; it should be noted that the solvation of Cr­(CO)_5_ is reported to occur in ca. 1 ps,^30^ although a recent study has a time constant of 8 ps for this event.^33^

On longer time scales, the incorporation of a PPh_3_ ligand into the coordination sphere of the manganacyclic complexes [Mn­(inpy)­(CO)_4–*n*_(PPh_3_)_*n*_] (*n* = 0, 1) appears to only have a negligible effect on the migratory insertion of the PhC_2_H in the Mn–C bond: a key step in Mn-catalyzed C–H bond functionalization reactions.

## Experimental Section

Detailed information about the materials, synthetic procedures, analytical methods, and computational chemistry is provided in the Supporting Information.

### Synthesis of [Mn­(inpy)­(CO)_4_], **2**

This compound was prepared using a modification of a literature procedure.^12^ An oven-dried Schlenk flask was cooled under a vacuum and refilled with N_2_. To this, MnBn­(CO)_5_ (143 mg, 0.5 mmol, 1 equiv) and **5** (97 mg, 0.5 mmol, 1 equiv) were added. The flask was evacuated and refilled with N_2_ five times (Note: MnBn­(CO)_5_ is susceptible to sublimation, so the flask was evacuated to just below 1 mbar, then returned to N_2_ refill). Toluene (20 mL) was added to the flask, which was covered in foil and heated at 100 °C and monitored by solution-state IR sampling. On completion of the reaction (disappearance of the MnBn­(CO)_5_ band at 2107 cm^–1^), the mixture was allowed to cool to room temperature and then filtered through Celite. The filtrate was transferred to a round-bottomed flask and dried under reduced pressure, affording the final product **2** (161 mg, 89%).

^13^C NMR (151 MHz, CDCl_3_) δ 218.98, 213.37, 211.21, 166.37, 157.00, 153.90, 140.10, 137.79, 136.51, 121.97, 120.10, 118.13, 117.86, 117.64, 110.72, 110.63; ^1^H NMR (400 MHz, CDCl_3_) δ 8.46 (ddd, *J* = 5.7, 1.7, 0.8 Hz, 1H), 7.92 (ddd, *J* = 8.6, 1.5, 0.8 Hz, 1H), 7.87 (ddd, *J* = 8.6, 7.0, 1.7 Hz, 1H), 7.77 (dq, *J* = 8.2, 0.9 Hz, 1H), 7.50 (ddd, *J* = 7.6, 1.4, 0.7 Hz, 1H), 7.31–7.08 (m, 4H), 6.94 (ddd, *J* = 7.1, 5.7, 1.5 Hz, 1H), 6.84 (d, *J* = 0.8 Hz, 1H). MS (LIFDI+): Calculated for C_17_H_9_N_2_O_4_Mn [M]^+^ 359.99373, Found 359.99545; IR (solution, toluene) ν_CO_ 1945, 1986, 1999, and 2083 cm^–1^; UV/vis (solution, toluene): ε at 400 nm = 1.23 mM^–1^ cm^–1^.

### Synthesis of [Mn­(inpye)­(CO)_3_(PPh_3_)], **3**

An oven-dried Schlenk flask was cooled under a vacuum and refilled with Ar three times. To this, **5** (95 mg, 0.49 mmol, 1 equiv) and **4** (250 mg, 0.49 mmol, 1 equiv) were added. The flask was evacuated and refilled with Ar once more. The stopper was replaced with a suba-seal, and dicyclohexylamine (0.2 mL, 2 equiv) and 1,2-dichloroethane (2 mL) were added via a syringe. The flask was covered in foil and heated at 80 °C overnight. On completion of the reaction, the mixture was allowed to cool to room temperature and transferred to a round-bottomed flask. The volume was reduced under reduced pressure, taking care to exclude light. To this, excess hexane was added, and the flask was placed in a freezer for several days, resulting in a yellow precipitate, which was isolated by suction filtration. Washing the solid with hot hexane afforded **3** (243 mg, 83%).

An adequate quality ^1^H NMR spectrum of **3** could not be obtained due to significant peak broadening presumably as a result of trace paramagnetic impurities; ^13^C NMR (151 MHz, CDCl_3_) δ 225.58 (d, *J*_PC_ = 21.6 Hz), 219.05 (d, *J*_PC_ = 24.6 Hz), 218.58 (d, *J*_PC_ = 17.9 Hz), 178.39 (d, *J*_PC_ = 24.3 Hz), 156.63, 153.86, 138.59, 137.68, 137.25 (d, *J*_PC_ = 3.8 Hz), 133.64 (d, *J*_PC_ = 9.6 Hz), 132.96 (d, *J*_PC_ = 34.2 Hz), 129.74, 129.72, 128.17 (d, *J*_PC_ = 8.8 Hz), 121.56, 119.10, 118.45 (d, *J*_PC_ = 5.3 Hz), 117.75, 116.98, 110.66, 110.03.; ^31^P NMR (162 MHz, CDCl_3_) δ 53.4; IR (solution, toluene) ν_CO_ 1900, 1932, and 2009 cm^–1^; MS (LIFDI+): Calculated for C_34_H_24_MnN_2_O_3_P [M]^+^ 594.09050, Found 594.09273; UV/vis (solution, toluene): ε at 400 nm = 2.62 mM^–1^ cm^–1^.

### Synthesis of [MnBr­(CO)_4_(PPh_3_)], **4**

This compound was prepared using the literature procedure.^34^ To a 25 mL foiled round-bottomed flask, MnBr­(CO)_5_ (1 g, 3.64 mmol), triphenylphosphine (954 mg, 3.64 mmol, 1 equiv), and 10 mL 1,2-dichloroethane were added. A layer of nitrogen was established using a suba-seal and balloon, and the mixture was stirred in an oil bath at 70 °C for 4 h. The reaction mixture was cooled to room temperature before drying under reduced pressure. IR and ^31^P NMR spectroscopic analysis showed quantitative conversion to **4**.

An adequate quality of ^1^H NMR spectrum of **4** could not be obtained due to significant peak broadening, presumably as a result of trace paramagnetic impurities ^13^C NMR (150 MHz, CDCl_3_) δ: 216.4, 211.6, 210.6, 133.6, 132.5, 130.9, 128.7; ^31^P NMR (162 MHz, CDCl_3_) δ 41.01; IR (solution, toluene) ν_CO_ 1957, 2003, 2020, and 2088 cm^–1^; MS (LIFDI+): Calculated for C_22_H_15_O_4_PMnBr [M]^+^ 507.92663, Found 507.92803; UV/vis (solution, toluene): ε at 400 nm = 0.70 mM^–1^ cm^–1^.

### Synthesis of 1-(Pyridin-2-yl)-1*H*-indole, **5**

An oven-dried round-bottomed Schlenk flask with a glass stopper was cooled under vacuum and then refilled with argon. Under a positive flow of argon, 1*H*-indole (2.81 g, 24 mmol, 1.2 equiv) and potassium hydroxide (crushed pellets, 2.36 g, 42 mmol, 2.1 equiv) were added. The flask was then evacuated and refilled with argon twice. Under a positive flow of argon, 25 mL of dimethyl sulfoxide was added using a funnel, followed by rapid replacement of the stopper. The mixture was stirred in a preheated oil bath at 135 °C for 1 h. The stopper was replaced with a suba-seal, and 2-bromopyridine (1.9 mL, 1.0 mmol, 1 equiv) was added using a syringe. The stopper was replaced, and the flask was sealed off from the argon flow and stirred for 48 h at 135 °C. On completion of the reaction, the solution was cooled to room temperature and quenched with 80 mL of EtOAc. The organic layer was separated and washed with 30 mL H_2_O, 30 mL NaOH (aq., 2 M), and 30 mL brine. The aqueous layer was washed with 30 mL of EtOAc. The organic layers were combined, dried over MgSO_4_, filtered, and concentrated under reduced pressure to yield an orange oil. Column chromatography using 1:8:1 diethyl ether/hexanes/triethylamine (preparation of the column was carried out with 10% diethyl ether in hexanes) yielded a colorless oil, which could be chilled in a freezer to a white solid (2.25 g, 58%). The characterization data were in agreement with the literature.^35^

^1^H NMR (400 MHz, CDCl_3_) δ 8.58 (ddd, J = 4.9, 2.0, 0.9 Hz, 1H), 8.23 (dq, *J* = 8.3, 0.8 Hz, 1H), 7.82 (ddd, *J* = 8.3, 7.3, 1.9 Hz, 1H), 7.74 (d, *J* = 3.5 Hz, 1H), 7.68 (ddt, *J* = 7.8, 1.3, 0.7 Hz, 1H), 7.50 (dt, *J* = 8.2, 0.9 Hz, 1H), 7.32 (ddd, *J* = 8.4, 7.0, 1.3 Hz, 1H), 7.27–7.13 (m, 2H), 6.73 (dd, *J* = 3.5, 0.8 Hz, 1H).^13^C NMR (101 MHz, CDCl_3_) δ 152.77, 149.26, 138.67, 135.35, 130.71, 126.26, 123.41, 121.55, 121.38, 120.33, 114.84, 113.28, 105.81; MS (ESI+): Calculated for C_13_H_12_N_2_ [M + H]^+^ 195.0917, Found 195.0916.

## Supplementary Material





## Data Availability

The data supporting this research is available for download from the research data repository of the University of York at https://doi.org/10.15124/176d779d-d2c9-4b52-bc07-105392c016ab.

## Notes

Collated coordinates and energies for computational structures (XYZ)
NMR spectra for compounds **2–5**; details of X-ray crystallography data collection and refinement; computational chemistry methods and further discussion. Details of the time-resolved spectroscopy experiment and data collection (PDF)
