# Supplementary material for: Probing Mn Precatalyst Activation through Time-Resolved Spectroscopy: A Quantitative Evaluation of the Effects of CO and PPh3 as Coligands on Ultrafast Dynamics and C–C Bond Formation
Source: Inorg Chem. 2025 Aug 12;64(33):16768–80. doi: 10.1021/acs.inorgchem.5c01443 (PMC12381853; doi:10.1021/acs.inorgchem.5c01443)
Supplement: Supplementary file 2 [file ic5c01443_si_002.pdf]

Supporting Information

Probing Mn pre-catalyst activation through time-resolved spectroscopy: a quantitative evaluation of the effects of CO and PPh_3 as co-ligands on ultra-fast dynamics and C–C bond formation.

Benjamin R. O'Donoghue,[a,b] Stefan Flesch,[a] Eimear Courtney,[b] Shweta Choudhary,[a] Jonathan B. Eastwood,[a] Katrina Mackey,[b] Leticia M. Pardo,[b] Ian P. Clark,[c] Partha Malakar,[c] Gregory M. Greetham.[c] Adrian C. Whitwood,[a] Richard J. Gammons,[a] Gerard P. McGlacken,*,[b] Ian J. S. Fairlamb,*,[a] and Jason M. Lynam*,[a]

a. Department of Chemistry, University of York, Heslington, York, YO10 5DD, UK.

b. School of Chemistry, Analytical & Biological Chemistry Facility, University College Cork, Cork, T12 YN60, Ireland.

c. Central Laser Facility, STFC Rutherford Appleton Laboratory, Harwell Science and Innovation Campus, Didcot, Oxfordshire, OX11 0QX, UK.

Corresponding Author E-mail Addresses

Gerard P. McGlacken - G.McGlacken@ucc.ie Ian J. S. Fairlamb – ian.fairlamb@york.ac.uk

Jason M. Lynam – Jason.lynam@york.ac.uk

Contents

1. General information and procedures 2
2. Single Crystal X-ray Data 5
3. Computational Chemistry 7
   3.1. Methodology 7
   3.2. Potential role for agostic complexes in the photochemistry of **3** 7
4. NMR Spectra 8
   4.1. Mn(1-(pyridin-2-yl)-1*H*-indole)$(CO)_4$, **2** 8
   4.2. Mn(1-(pyridin-2-yl)-1H-indole)$(CO)_3(PPh_3)$, **3** 10
   4.3. $MnBr(CO)_4(PPh_3)$, **4** 12
   4.4. 1-(pyridin-2-yl)-1*H*-indole, **5** 14
5. References 16

1. General information and procedures

Solvents and Reagents

Commercial chemicals were purchased from (Acros Organics, Alpha Aesar, Apollo Scientific, Fisher Scientific, Fluorochem, Insight Biotechnology, Merck Life Science, Sigma-Aldrich, Strem Chemicals UK, or Tokyo Chemical Industry UK) and were used without further purification unless otherwise stated. Dry toluene was purchased from Acros Organics, stored over 4 Å molecular sieves and under an atmosphere of N_2 or collected from a Pure Solv MD-7 solvent system and stored in oven dried ampoules under an atmosphere of N_2. Room-temperature (RT) typically refers to 21 °C, with an upper and lower limit of 16−23 °C recorded.

Chromatography

Thin-layer chromatography (TLC) was conducted using Merck aluminium-backed 5554 silica plates. Visualisation of spots was achieved *via* irradiation (254 nm), or sequential staining with potassium permanganate followed by heating. Flash column chromatography was carried out following the procedure reported by Still *et al.*,[1] using Fluorochem silica gel 60 (particle size 40−63 µm), with the solvent system stated in the specific procedure

Infrared Spectroscopy

Infrared spectra for characterisation were obtained using a Bruker Vertex 80 FTIR spectrometer.

UV−Visible spectroscopy

UV−Visible spectra were recorded with a Jasco V−560 spectrometer using Quartz cuvettes.

Nuclear Magnetic Resonance Spectroscopy

Solution phase ^{1}H, ^{31}P, and ^{13}C NMR analysis were recorded on a JEOL ECX400 or JEOL ECS400 spectrometer (400, 100, and 162 MHz for ^{1}H, ^{13}C, and ^{31}P respectively) at 298 K at the University of York. The ^{13}C analysis for complexes **3** and **4** were recorded on Bruker Advance 600 operating at 151 MHz at University College Cork. The ^{13}C analysis for complex **2** was recorded on a Bruker ___ 600 operating at 151 MHz at the University of York. ^{13}C and ^{31}P NMR spectra were recorded with ^{1}H decoupling. Spectra were processed in MestReNova software version 14.0.0-23239. In ^{1}H spectra, coupling constants were quoted with ± 0.5 Hz. Chemical shifts are reported in ppm and referenced to the residual non-deuterated solvent.

Residual $CHCl_3$ in chloroform-d: ^{1}H: $CHCl_3$ 7.26 ppm, ^{13}C: $CHCl_3$ 77.36 ppm

^{1}H NMR peaks are reported to two decimal places, whereas ^{31}P and ^{13}C are reported to one decimal place.

Mass Spectrometry

ESI MS spectra were measured using a Bruker Daltronics micrOTOF MS, Agilent series 1200LC with electrospray ionization. Liquid Injection Field Desorbtion Ionisation (LIFDI) mass spectrometry was carried out using a Waters GCT Premier MS Agilent 7890A GC. Data were quoted as a mass to charge ratio (m/z) in Daltons and relative intensity in parenthesis. High resolution mass spectra (HRMS) are reported within 5 ppm error of the theoretical value unless stated otherwise.

Single Crystal X-ray Diffraction experiments.

Single crystals were crystalised from a suitable solvent system noted in the relevant synthetic procedure. An appropriate crystal was selected and [oil on 200 micrometre micromount] on a Rigaku Synergy-S X-ray diffractometer equipped with a copper source (λ = 1.54184 Å), with a Hypix-Arc 100 detector. The crystal was kept at 110.00(10) K during data collection using an Oxford Cryosystems Cryostream 1000. Using Olex2,[2] the structure was solved with the SHELXT[3] structure solution program using Intrinsic Phasing and refined with the SHELXL[4] refinement package using Least Squares minimisation.

Time-Resolved Multiple Probe Spectroscopy (TR^{M}PS)

TRIR measurements were carried out at the LIFEtime facility using the TR^{M}PS technique at the Central Laser Facility (Science and Technology Facility Council Rutherford Appleton Laboratories).[5,6] The experiments were driven by a 100 kHz repetition rate Yb:KGW amplifier (Pharos) as a pump source, producing 15 W, 260 fs pulses at 1030 nm. The laser output was used to drive a BBO-based 515 nm pumped optical parametric amplifier (OPA). The pump beam was collimated, travelled along a programable optical delay line (0-16 ns 1200 mm long double pass), then focused onto the sample. The probe beam sources were from a 100 kHz repetition rate Yb:KGW amplifier (Pharos) producing 6W, 180 fs pulses at 1030 nm, driving two 3 W BBO/KTA based OPAs. The two Pharos sources shared a 80 MHz oscillator, allowing pump-probe delay steps of 12.5 ns. The probe beam was split to provide probe and reference pulses. The probe beams were collimated, synchronised by a fixed optical delay, and focused by a gold parabolic mirror onto the sample. The three beams were overlapped on the sample using a 50 µm pinhole. The probe beams were measured by two separate 128-element detectors. To go beyond 12.5 ns, subsequent seed pulses can be selected from the 80 MHz oscillator.

Data were collected using two different pump repetition rates. For pump-probe delays ranging from 1 ps to 988.5 µs a pump repetition rate of 1 kHz was employed. Samples were prepared in an oven

dried amberised Duran bottle. Approximately 12 mg (**3**) or 7 mg (**2**) of manganese complex was added and dissolved in 10 mL of anhydrous solvent from a newly opened sure-seal bottle. The system was then sparged (N_2, Ar, or air) for 10 minutes with solution pumping around the system. For the duration of the experiment, the Duran flask was sealed while under a positive pressure of sparge gas. The Duran bottle was connected *via* PTFE tubing to a Harrick cell with a spacer (200 μm unless stated otherwise), with a peristaltic pump used to flow the solution through the system. During experiments, the Harrick cell was rastered in two dimensions, to prevent excitation of photoproducts. Following an experiment all solution was pumped from the system, 3 × 10 mL of new solvent pumped around to clean the kit, and dried using a positive pressure of N_2 for 10 minutes.

Time-resolved $TR^{M}PS$ spectra were processed by means of a self-written software employing the program language *Julia*. The raw data sets were subjected to a correction of the zero point of the time axis, averaging of spectra recorded at delays longer than 10 microseconds onto a logarithmic axis containing fewer points, a correction of global artefacts by subtraction of signals occurring at delays < -10 ps, linear baseline corrections and a calibration of the spectral axis using the known absorption frequencies of the starting complexes **2** and **3**. Additionally, in order to cover the entire, relevant spectral region, two partially overlapping spectral windows were recorded and combined using the same software. The resulting data were then analysed in OriginPro 2019b (64-bit) 9.6.5.169 (Academic) software. Where data sets were particularly noisy, early time points after the first trump were deleted and up to a 20-point average of data points applied. Kinetic fits were performed with appropriate ExpGro, ExpDec and ExpGroDec functions and values were quoted with in the format of XX ± XX indicating the 95% confidence limits of values obtained from exponential fits. The suitability of kinetic fits was then assessed using the built-in residual plots produced by OriginPro software.

2. Single Crystal X-ray Data

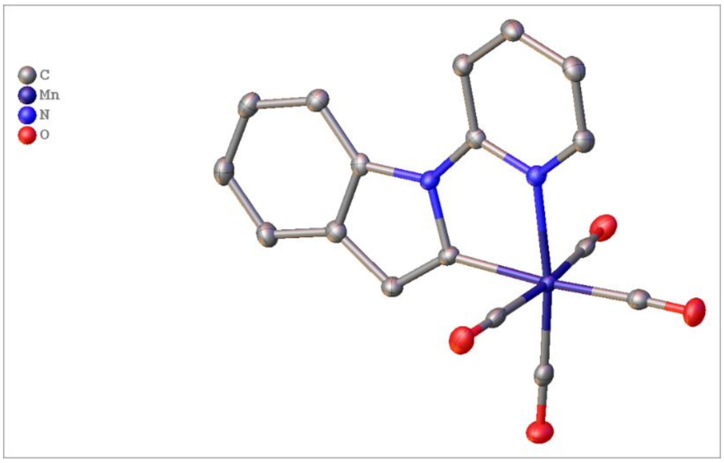

Data collected, solved and refined by Adrian C Whitwood

Table S1 Crystal data and structure refinement for Compound 2

CCDC Deposition Number	2433013
Empirical formula	$C_{17}H_9MnN_2O_4$
Formula weight	360.20
Temperature/K	110.00(10)
Crystal system	monoclinic
Space group	$P2_1/n$
a/Å	11.05862(17)
b/Å	12.62655(18)
c/Å	11.58127(18)
α/°	90
β/°	115.1278(19)
γ/°	90
Volume/Å^3	1464.08(4)
Z	4
ρ_{calc} g/cm^3	1.634
μ/mm^{-1}	7.567
F(000)	728.0
Crystal size/mm^3	0.26 × 0.175 × 0.059
Radiation	Cu Kα (λ = 1.54184)
2Θ range for data collection/°	9.268 to 136.462
Index ranges	-13 ≤ h ≤ 11, -11 ≤ k ≤ 15, -13 ≤ l ≤ 13
Reflections collected	9481
Independent reflections	2678 [R_{int} = 0.0292, R_{sigma} = 0.0268]
Data/restraints/parameters	2678/0/254
Goodness-of-fit on F^2	1.064
Final R indexes [I>=2σ (I)]	R_1 = 0.0241, wR_2 = 0.0635
Final R indexes [all data]	R_1 = 0.0251, wR_2 = 0.0640
Largest diff. peak/hole / e Å^{-3}	0.26/-0.26

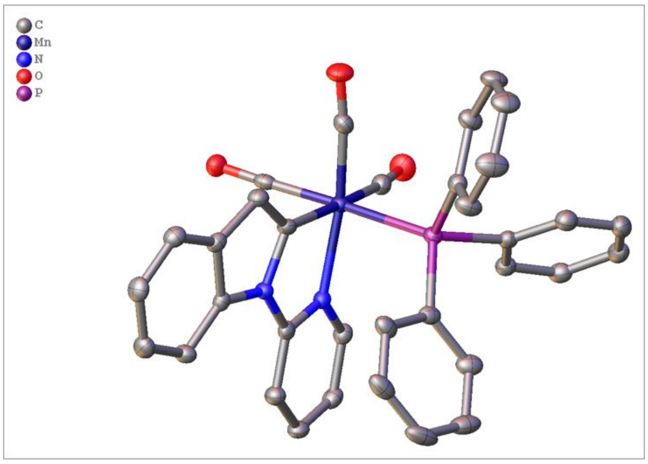

Data Collected, Solved and Refined by Richard J Gammons

Table S2 Crystal data and structure refinement for Compound 3

CCDC Deposition Number	2433014
Empirical formula	$C_{34}H_{24}MnN_2O_3P$
Formula weight	594.46
Temperature/K	110.00(10)
Crystal system	monoclinic
Space group	I2/a
a/Å	22.4180(3)
b/Å	8.47390(10)
c/Å	28.8971(4)
α/°	90
β/°	100.4100(10)
γ/°	90
Volume/Å^3	5399.16(12)
Z	8
ρ_{calc} g/cm^3	1.463
μ/mm^{-1}	4.862
F(000)	2448.0
Crystal size/mm^3	0.212 × 0.041 × 0.018
Radiation	Cu Kα (λ = 1.54184)
2Θ range for data collection/°	6.22 to 136.524
Index ranges	-12 ≤ h ≤ 26, -10 ≤ k ≤ 10, -34 ≤ l ≤ 34
Reflections collected	8650
Independent reflections	8650 [R_{sigma} = 0.0378]
Data/restraints/parameters	8650/0/371
Goodness-of-fit on F^2	1.048
Final R indexes [I>=2σ (I)]	R_1 = 0.0383, wR_2 = 0.0972
Final R indexes [all data]	R_1 = 0.0529, wR_2 = 0.1047
Largest diff. peak/hole / e Å^{-3}	0.31/-0.31

The crystal was a non-merohedral twin which was modelled using two components in a refined ratio of 0.7573: 0.2427(7).

3. Computational Chemistry

3.1. Methodology

Calculations to determine the relative energies of intermediates were performed using the TURBOMOLE V7.8.1 package using the resolution of identity (RI) approximation.[7-14] Initial optimisations were performed at the (RI-)BP86/SV(P) level, followed by frequency calculations at the same level. All minima were confirmed as such by the absence of imaginary frequencies. Single-point energies were then performed on the (RI-)BP86/SV(P) optimised geometries using the hybrid PBE0 functional and the flexible def2-TZVPP basis set. Energies, xyz coordinates and the first 50 lines of the vibrational spectra are presented. Solvation effects were modelled using COMSO[15] using the dielectronic constant of 2.38 for toluene and energies were corrected for dispersion using Grimme's D3-method[16] with Becke-Johnson dampening.[17]

3.2. Potential role for agostic complexes in the photochemistry of **3**

The TRIR data presented in Figure 4 demonstrate that **3** undergoes selective CO, rather than PPh_3, dissociation. This results in the formation of a putative complex [Mn(inpy)$(CO)_2$(PPh_3)] which has a formal vacant coordination site. In the manuscript, it is argued that this species is rapidly solvated by toluene to form **9**. It was also considered that [Mn(inpy)$(CO)_2$(PPh_3)] could achieve an 18-electron configuration through the formation of a C–H agostic interaction from one of the phenyl groups on the PPh_3 ligand. Although it was possible to obtain these structures as minima on the potential energy surface, a quantitative evaluation of their relative energy compared to a coordinated toluene ligand was fraught with difficulty and marked computational method effects were observed. For example, the results when attempting to optimise structure **S1** were markedly different if the optimisation method used was BP86/SV(P) with or without an applied D3(BJ) dispersion correction. When the correction was not applied during the optimisation, this state is dissociative in toluene, whereas when the correction was used, then a bound toluene complex was obtained.

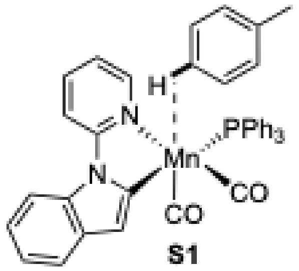

S1

Furthermore, the relative energies of the agostic and toluene complexes were profoundly influenced by the applied D3(BJ) correction. In brief, the application of the dispersion correction stabilised the toluene complex with respect to the corresponding agostic species. This is of course expected, but in this model the corresponding (favourable) dispersion interactions between the liberated and bulk toluene are not modelled which may skew the chemical picture. The available spectroscopic and computational data does appear to indicate that **9**(π) is formed in these reactions which of course by virtue of the phosphine and toluene ligands being mutually *trans*, excludes the possibility of a competing agostic interaction, at least in this geometric configuration.

4. NMR Spectra

4.1. Mn(1-(pyridin-2-yl)-1*H*-indole)(CO)$_4$, **2**

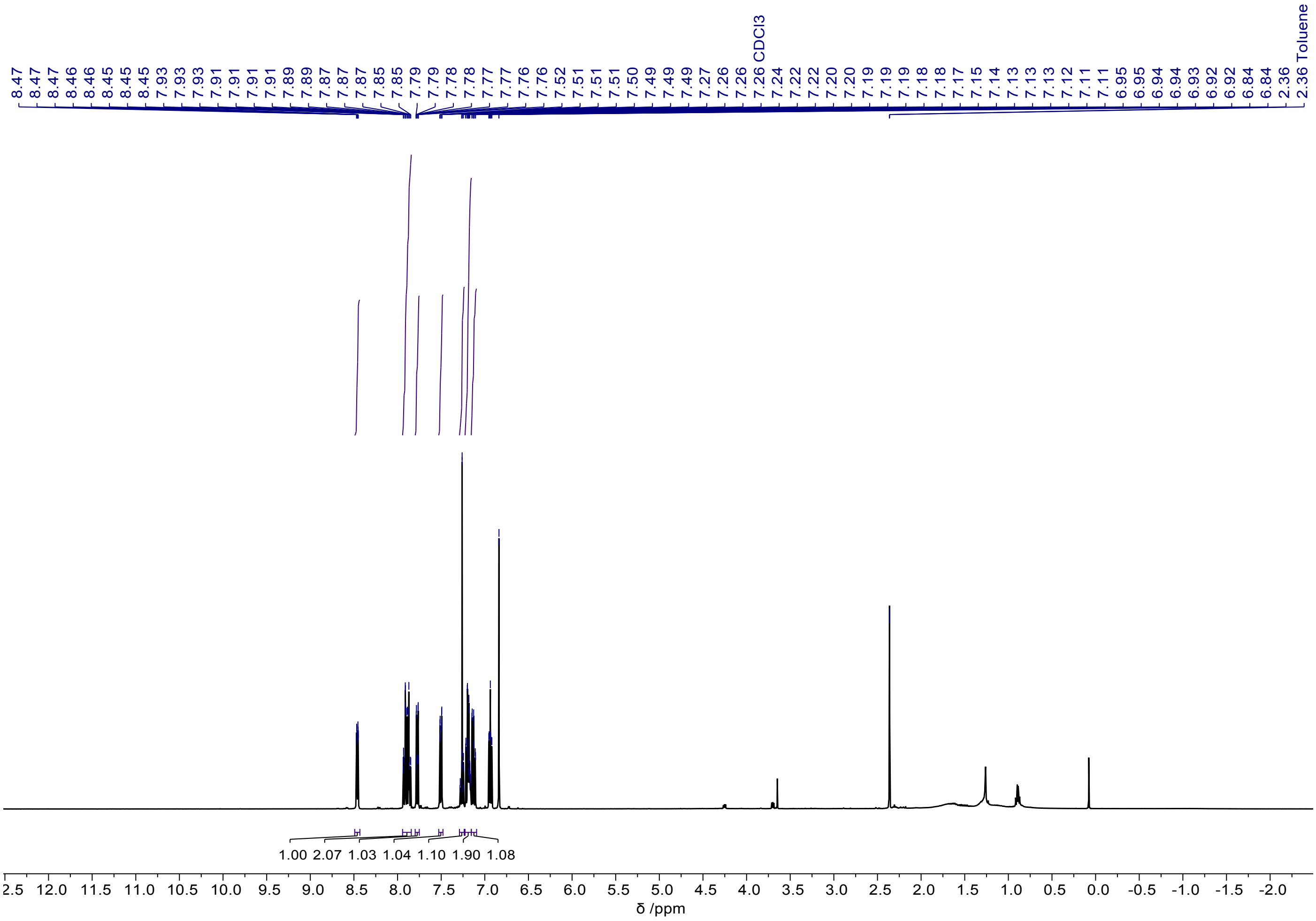

Figure S1. ^{1}H NMR spectrum of **2** recorded at 400 MHz in $CDCl_3$ solution at 298 K.

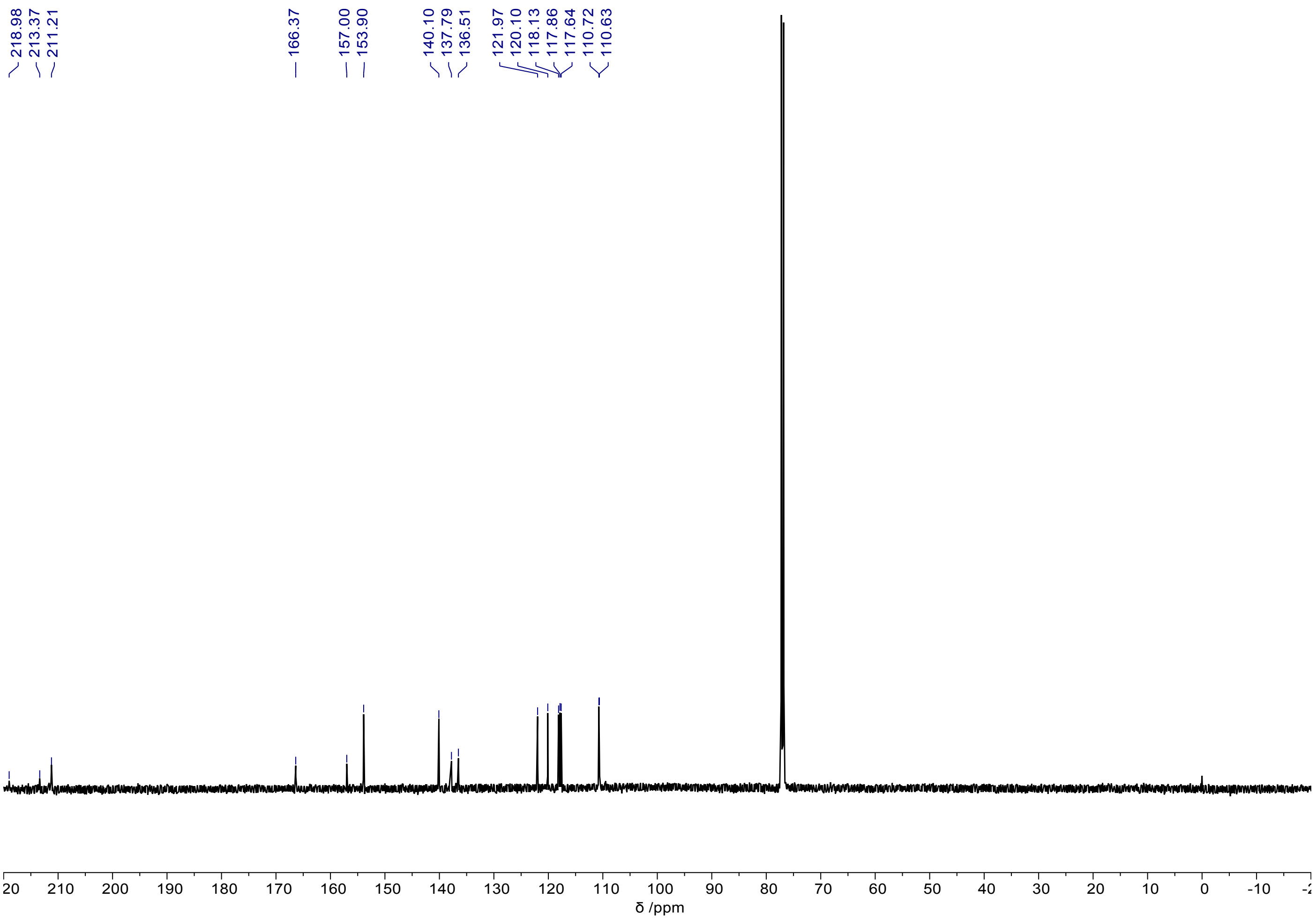

Figure S2. $^{13}C\{^{1}H\}$ NMR spectrum of **2** recorded at 161 MHz in $CDCl_3$ solution at 298 K.

4.2. Mn(1-(pyridin-2-yl)-1H-indole)$(CO)_3(PPh_3)$, **3**

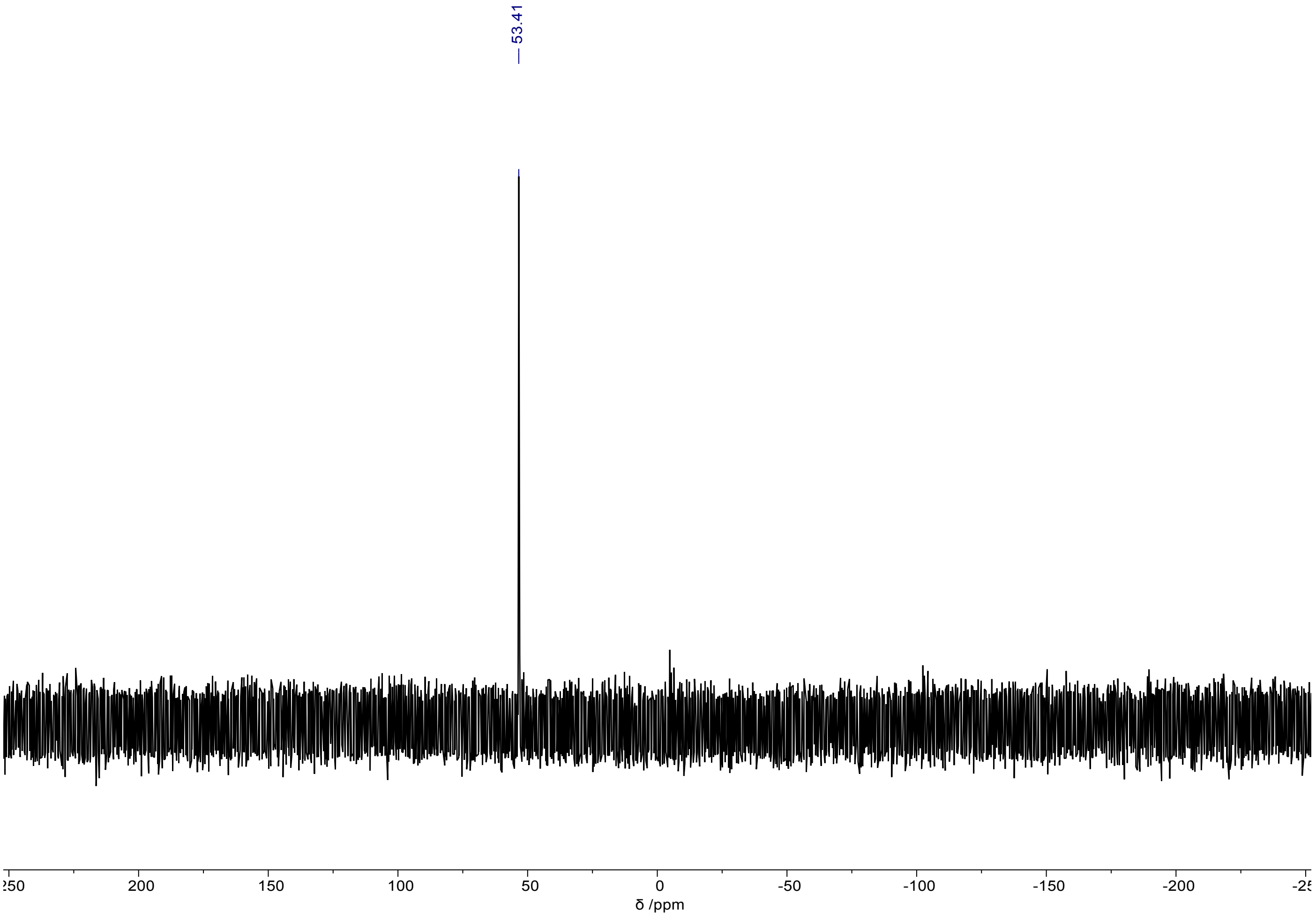

Figure S3. $^{31}P\{^1H\}$ NMR spectrum of **3** recorded at 162 MHz in $CDCl_3$ solution at 298 K.

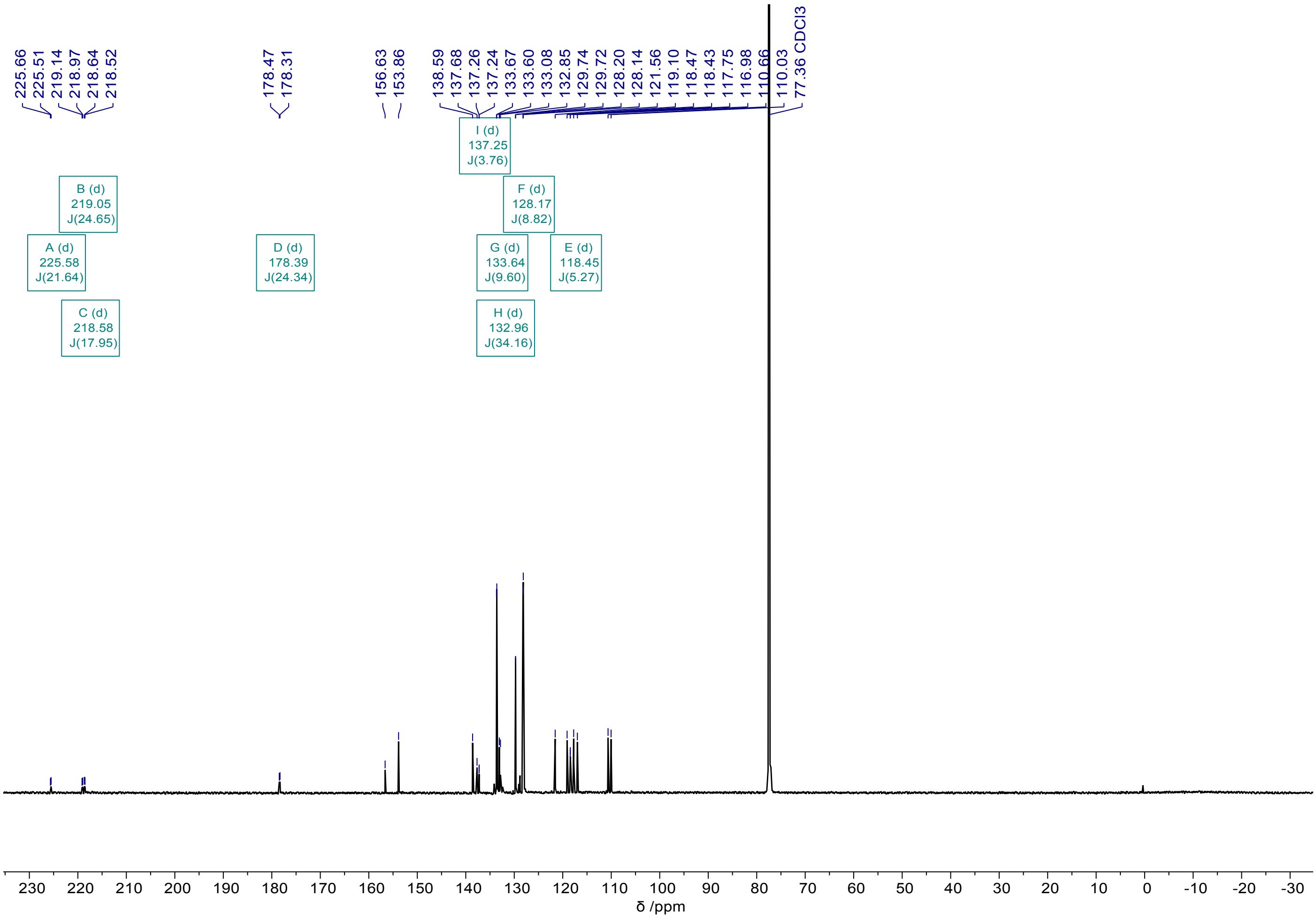

Figure S4. $^{13}C\{^{1}H\}$ NMR spectrum of **3** recorded at 161 MHz in $CDCl_3$ solution at 298 K.

4.3. MnBr(CO)$_4$(PPh$_3$), **4**

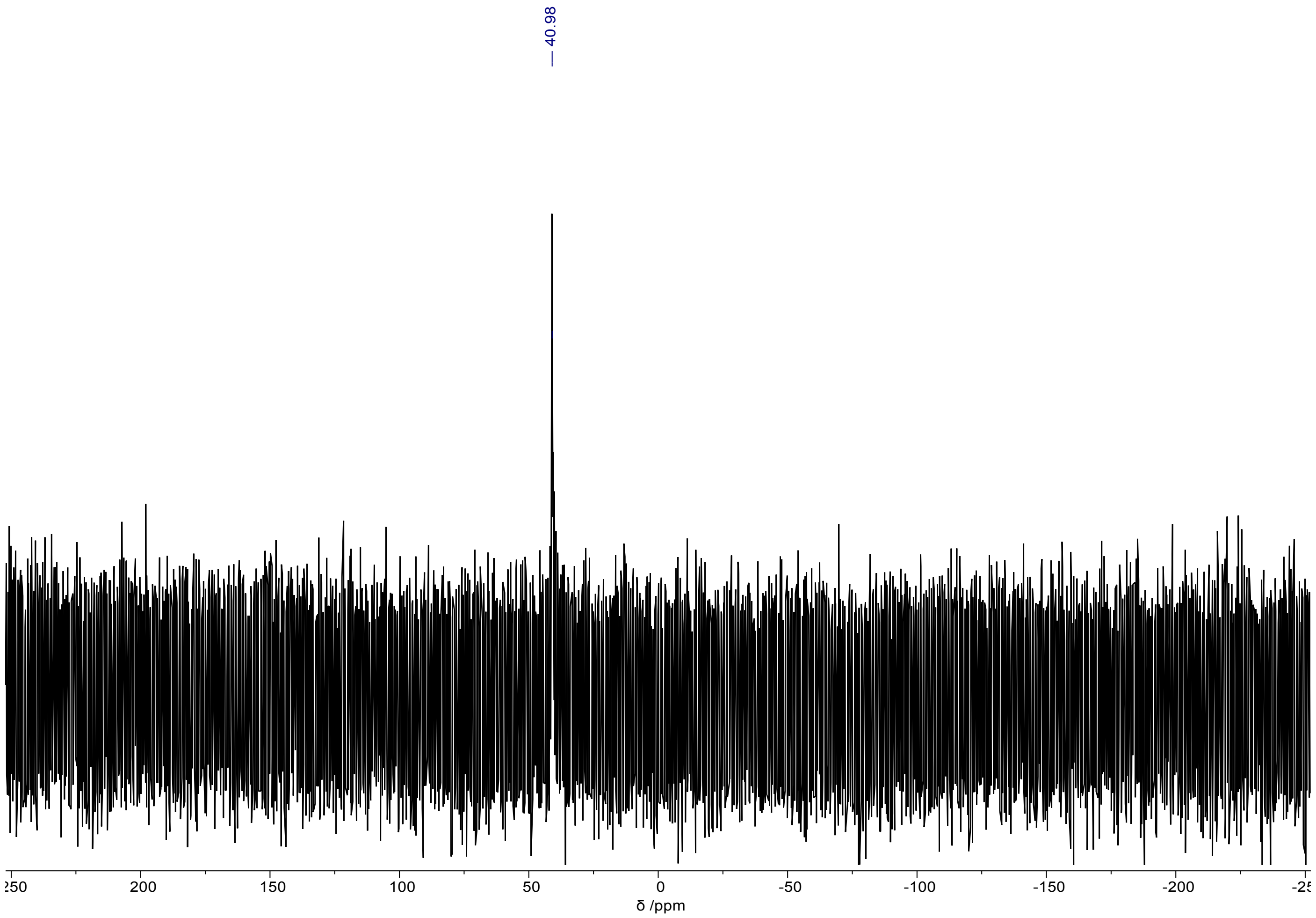

Figure S5. $^{31}P\{^1H\}$ NMR spectrum of **4** recorded at 162 MHz in $CDCl_3$ solution at 298 K.

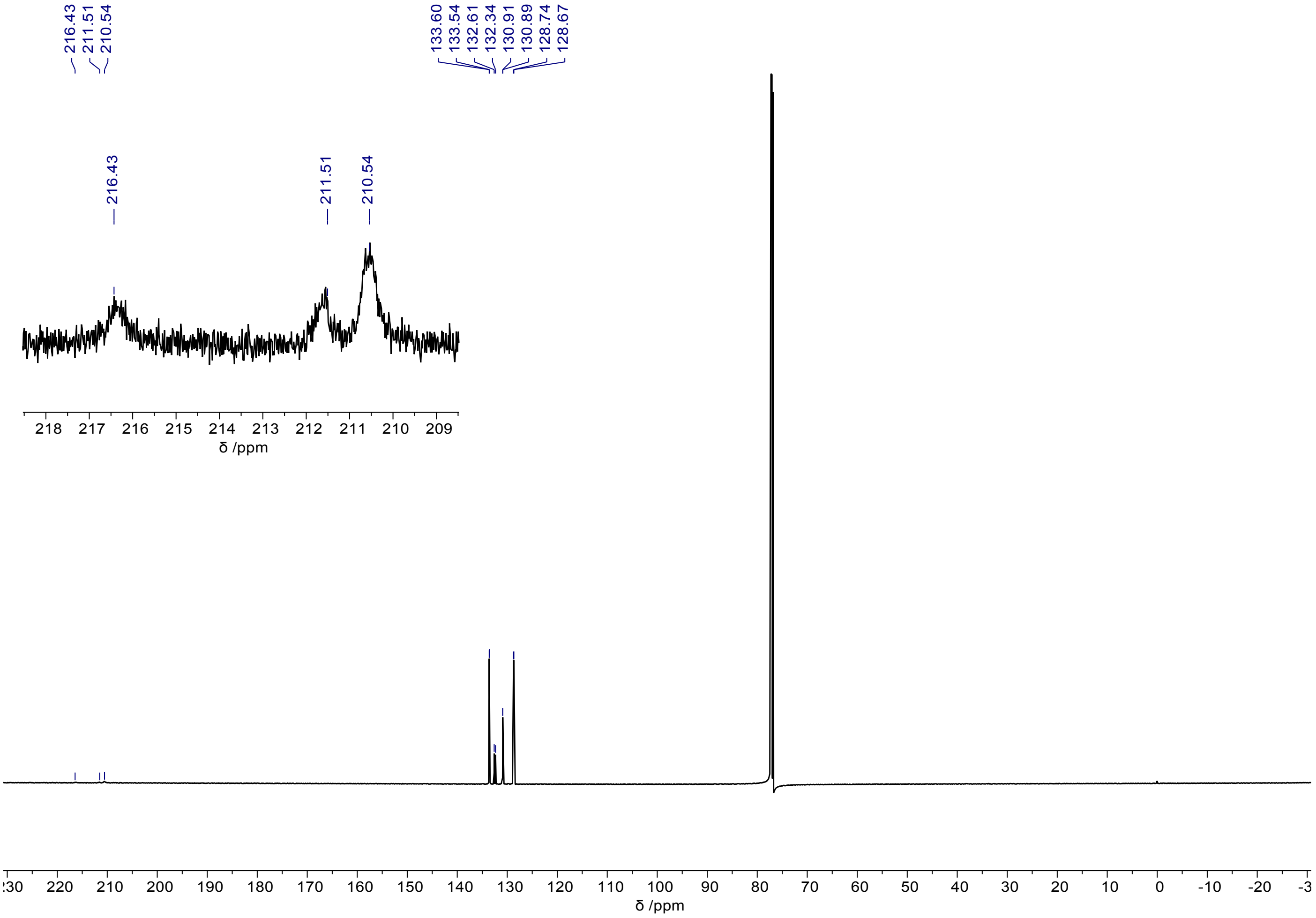

Figure S6. $^{13}C\{^{1}H\}$ NMR spectrum of **4** recorded at 161 MHz in $CDCl_3$ solution at 298 K, with an expansion of the region between ~ 209-218 ppm.

4.4. 1-(pyridin-2-yl)-1*H*-indole, **5**

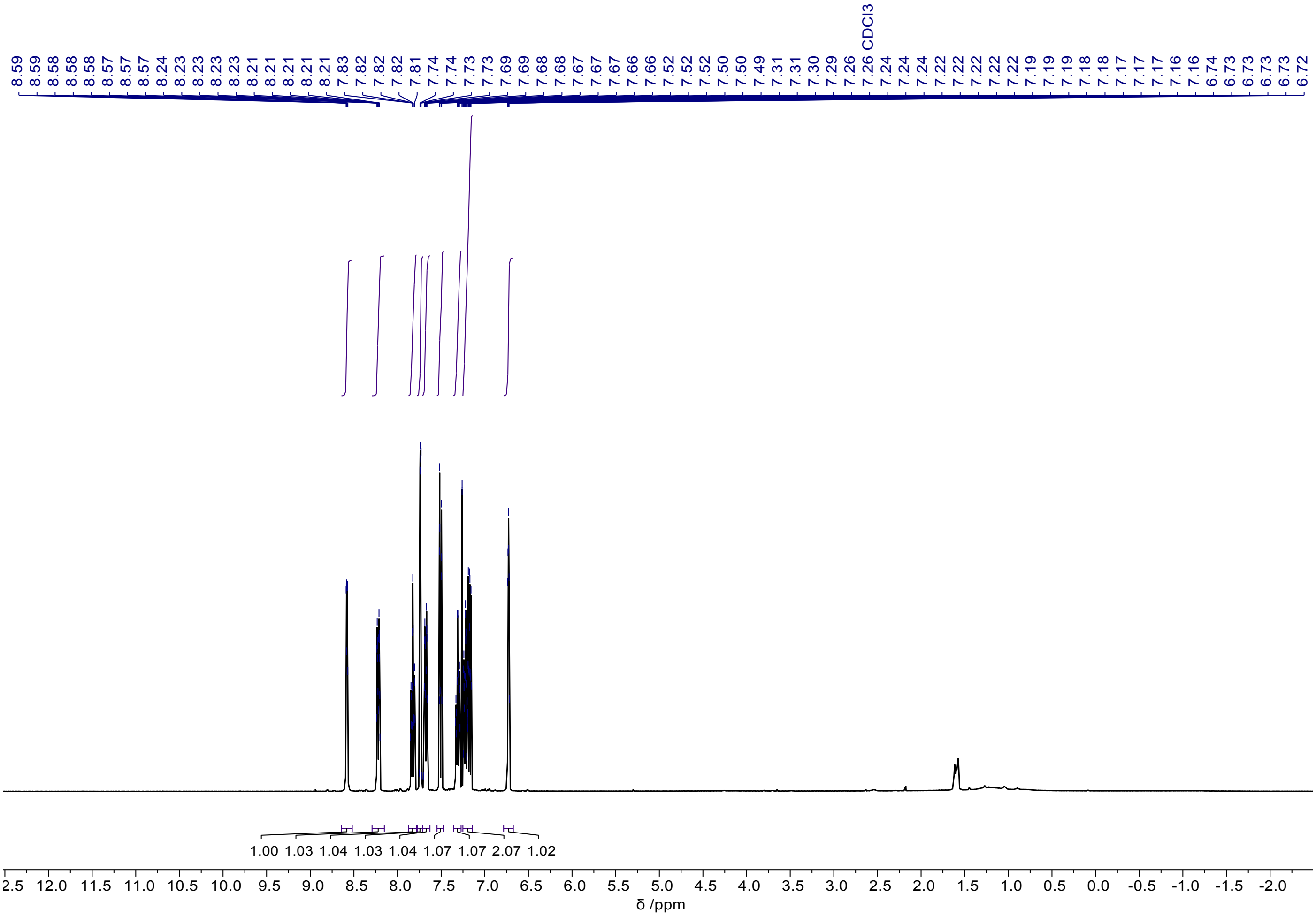

Figure S7. ^{1}H NMR spectrum of **5** recorded at 400 MHz in $CDCl_3$ solution at 298 K.

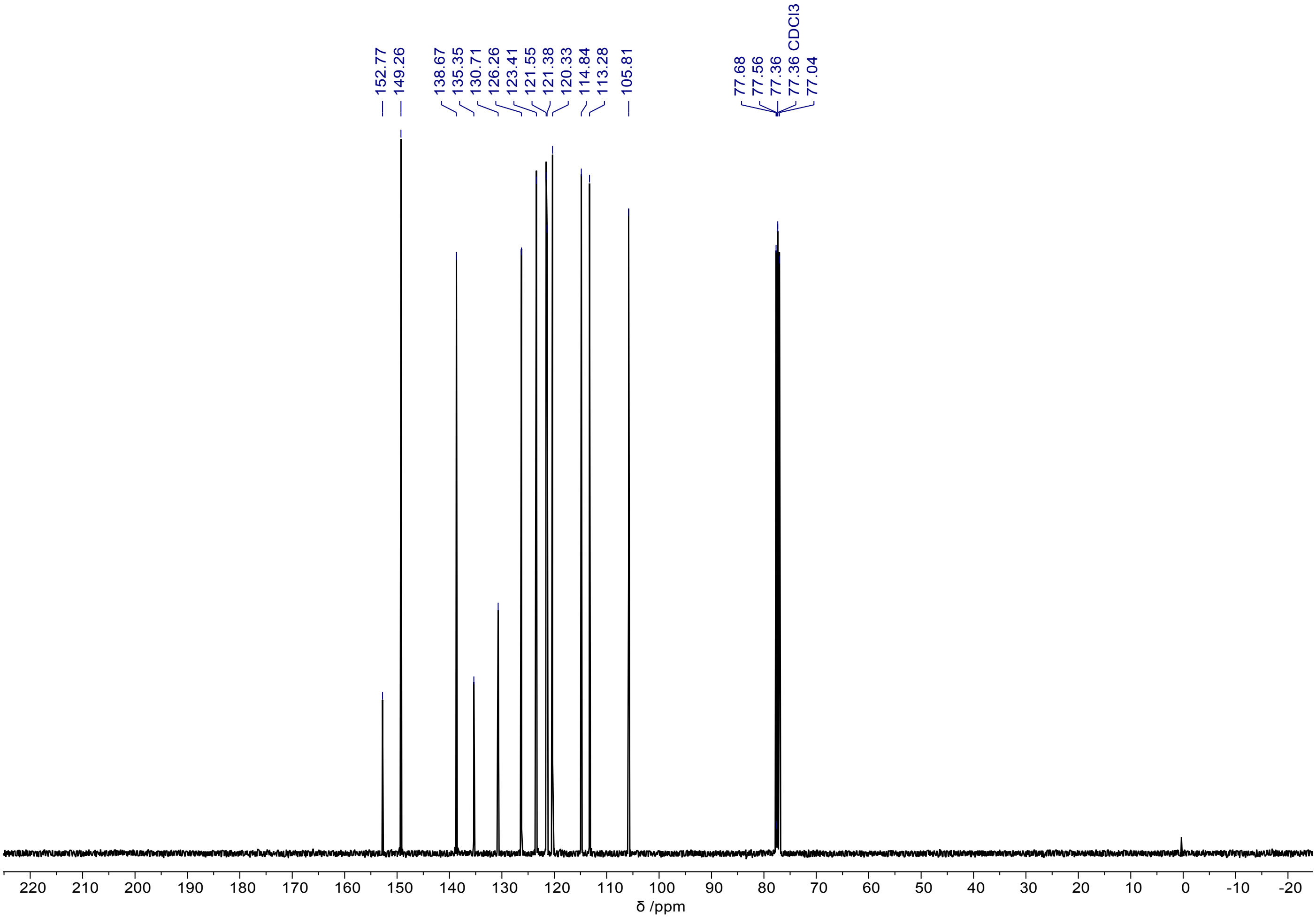

Figure S8. $^{13}C\{^{1}H\}$ NMR spectrum of **5** recorded at 161 MHz in $CDCl_3$ solution at 298 K.

5. References

1 Still, W. C.; Kahn, M.; Mitra, A. Rapid chromatographic technique for preparative separations with moderate resolution *J. Org. Chem*. **1978**, *43*, 2923–2925.

2 Dolomanov, O. v; Bourhis, L. J.; Gildea, R. J.; Howard J. A. K.; Puschmann, H. OLEX2: a complete structure solution, refinement and analysis program *J. Appl. Cryst*. **2009**, *42*, 339–341.

3 Sheldrick, G. M. SHELXT – Integrated space-group and crystal-structure determination *Acta Crystallogr.* **2015**, *A71*, 3–8.

4 Sheldrick, G. M. Crystal structure refinement with SHELXL *Acta Crystallogr*. **2015***, C71*, 3–8.

5 Greetham, G. M.; Sole, D.; Clark, I. P.; Parker, A. W.; Pollard, M. R.; Towrie, M. Time-resolved multiple probe spectroscopy *Rev. Sci. Instrum*. **2012**, *83*, 103107.

6 Greetham, G. M.; Donaldson, P. M.; Nation, C.; Sazanovich, I. V.; Clark, I. P.; Shaw, D. J.; Parker, A. W.; Towrie, M. *Appl. Spectrosc.* **2016**, *70*, 645-653.

7 Császár, P.; Pulay, P. Geometry optimization by direct inversion in the iterative subspace. *J. Mol. Struct.* **1984**, *114*, 31-34.

8 Ahlrichs, R.; Bär, M.; Häser, M.; Horn, H.; Kölmel, C. Electronic structure calculations on workstation computers: The program system turbomole. *Chem. Phys. Lett.* **1989**, *162*, 165-169.

9 Deglmann, P.; Furche, F.; Ahlrichs, R. An efficient implementation of second analytical derivatives for density functional methods. *Chem. Phys. Lett.*, **2002**, *362*, 511-518.

10 Deglmann, P., May, K., Furche, F. & Ahlrichs, R. Nuclear second analytical derivative calculations using auxiliary basis set expansions. *Chem. Phys. Lett.* **2004**, *384*, 103-107.

11 Eichkorn, K., Treutler, O., Öhm, H., Häser, M. & Ahlrichs, R. Auxiliary basis sets to approximate Coulomb potentials. *Chem. Phys. Lett.***1995**, *242*, 652-660.

12 Eichkorn, K., Weigend, F., Treutler, O. & Ahlrichs, R. Auxiliary basis sets for main row atoms and transition metals and their use to approximate Coulomb potentials. *Theor. Chem. Acc.* **1997**, *97*, 119-124.

13 Treutler, O. & Ahlrichs, R. Efficient molecular numerical integration schemes. *J. Chem. Phys.* **1995**, *102*, 346-354.

14 von Arnim, M. & Ahlrichs, R. Geometry optimization in generalized natural internal coordinates. *J. Chem. Phys.* **1999**, *111*, 9183-9190.

15 Klamt, A. & Schuurmann, G. COSMO: a new approach to dielectric screening in solvents with explicit expressions for the screening energy and its gradient. *J. Chem. Soc., Perkin Trans.* 2 **1993**, 799-805.

16 Grimme, S.; Antony, J.; Ehrlich, S.; Krieg, H. A consistent and accurate ab initio parametrization of density functional dispersion correction (DFT-D) for the 94 elements H-Pu *J. Chem. Phys.* **2010**, *132*, 154104.

17 Grimme, S.; Ehrlich, S.; and Goerigk, L. Effect of the damping function in dispersion corrected density functional theory *J. Comput. Chem.* **2011**, *32*, 1456.
